# Silver nanoparticles: Synthesis, medical applications and biosafety

**DOI:** 10.7150/thno.45413

**Published:** 2020-07-11

**Authors:** Li Xu, Yi-Yi Wang, Jie Huang, Chun-Yuan Chen, Zhen-Xing Wang, Hui Xie

**Affiliations:** 1 Department of Orthopedics, Xiangya Hospital, Central South University, Changsha, Hunan 410008, China.; 2 Movement System Injury and Repair Research Center, Xiangya Hospital, Central South University, Changsha, Hunan 410008, China.; 3 Xiangya Hospital of Central South University-Amcan Medical Biotechnology Co. Ltd. Joint Research Center, Changsha, Hunan 410008, China.; 4 Institute of Reproductive and Stem Cell Engineering, School of Basic Medical Science, Central South University, Changsha 410013, China.; 5 Department of Sports Medicine, Xiangya Hospital, Central South University, Changsha, Hunan 410008, China.; 6 Hunan Key Laboratory of Organ Injury, Aging and Regenerative Medicine, Changsha, Hunan 410008, China.; 7 Hunan Key Laboratory of Bone Joint Degeneration and Injury, Changsha, Hunan 410008, China.; 8 National Clinical Research Center for Geriatric Disorders, Xiangya Hospital, Central South University, Changsha, Hunan 410008, China.

**Keywords:** Silver nanoparticles, Silver Ångstrom particles, Synthesis, Antimicrobial, Anticancer, Toxicity, Mechanisms

## Abstract

Silver nanoparticles (AgNPs) have been one of the most attractive nanomaterials in biomedicine due to their unique physicochemical properties. In this paper, we review the state-of-the-art advances of AgNPs in the synthesis methods, medical applications and biosafety of AgNPs. The synthesis methods of AgNPs include physical, chemical and biological routes. AgNPs are mainly used for antimicrobial and anticancer therapy, and also applied in the promotion of wound repair and bone healing, or as the vaccine adjuvant, anti-diabetic agent and biosensors. This review also summarizes the biological action mechanisms of AgNPs, which mainly involve the release of silver ions (Ag^+^), generation of reactive oxygen species (ROS), destruction of membrane structure. Despite these therapeutic benefits, their biological safety problems such as potential toxicity on cells, tissue, and organs should be paid enough attention. Besides, we briefly introduce a new type of Ag particles smaller than AgNPs, silver Ångstrom (Å, 1 Å = 0.1 nm) particles (AgÅPs), which exhibit better biological activity and lower toxicity compared with AgNPs. Finally, we conclude the current challenges and point out the future development direction of AgNPs.

## Introduction

Silver and its compounds have been used for antibacterial and therapeutic applications for thousands of years 1, 2. Ancient Greeks and Romans used silverwares to store water, food, and wine to avoid spoilage. Hippocrates used silver preparations to treat ulcers and promote wound healing. Silver nitrate was also used for wound care and instrument disinfection. In 1852, Sims sutured the vesicovaginal fistulas caused by delivery with fine silver wires which significantly decreased infection. At the beginning of the 19th century, silver preparations were developed for wound infection and burn care. However, in the 1940s, the medical applications of silver gave way to the clinical introduction of antibiotics 1. With the abuse of antibiotics, bacterial resistance has become a worldwide problem especially since the 1980s, and silver began to receive attention again especially with the development of nanotechnology in the early of this century.

Nanomaterials (1-100 nm materials) have been attracting much attention in the past few decades in many fields such as biomedicine, catalysis, energy storage, and sensors, due to their unique physicochemical properties as compared to their bulk forms. Silver nanoparticles (AgNPs) have received special interest, especially in biomedicine. AgNPs are famous for their broad-spectrum and highly efficient antimicrobial and anticancer activities. Other biological activities of AgNPs have been also explored, including promoting bone healing and wound repair, enhancing the immunogenicity of vaccines 3, and anti-diabetic effects 4. Deciphering the biological mechanisms and potential cytotoxicity of AgNPs will facilitate their better medical applications.

Herein, we review the achievements of AgNPs in the past decade, especially focused on the past five years. This review intends to provide a valuable reference for researchers who are interested in the biomedical applications of AgNPs. The main contents include:

- Synthesis of AgNPs, including physical, chemical and biological synthesis methods;
- Medical applications of AgNPs, focusing on antimicrobial and anticancer properties and potential mechanisms, as well as other medical applications, including wound repair, bone healing, dental applications, vaccine adjuvant, antidiabetic agent, and biosensing;
- The potential toxicity of AgNPs, including potential damages of AgNPs to many systems and organs *in vivo*, including skin, eyes, respiratory system, hepatobiliary system, central nervous system, urinary system, immune system and reproductive system.

Numerous studies focus on the synthesis of AgNPs with controlled size and shape, and a variety of specific synthetic methods have been developed, including physical, chemical, and biological methods 5. The predominant processes of the physical methods are classified into two parts: mechanical and vapor-based processes 6. Conventional physical methods may involve mill, pyrolysis, and spark discharging 7. Physical synthesis can obtain AgNPs with uniform size distribution and high purity 8. Chemical synthesis is the most commonly used method to obtain AgNPs 8. This method involves reducing silver ions to silver atoms 9, and the process can be divided into two steps, nucleation and growth 10. Size- and shape-controlled AgNPs can be obtained by regulating the growth rate of nucleation. Besides reducing agents, capping agents and stabilizers also play important roles in obtaining AgNPs with good dispersion stability and uniform size distribution 11. In addition, external energy can synergistically synthesize AgNPs, such as microwave, light, heat, and sound 12-15. Although chemical synthesis methods of AgNPs are widely used, the toxicity and pollution caused by chemicals must be highlighted and more attention should be given. Compared with physical and chemical methods, the biological method proves an economical and environmental approach for AgNPs 8. Microorganisms include bacteria, fungi, and algae, as well as plant parts, include bark, peel, callus, leaves, flower, fruit, stem, seed, and rhizome are widely used in biological synthesis 16. The organics, like enzymes, alkaloids, phenolic compounds and terpenoids, are abundant in extracts of microorganisms and plants, which can be available to reduce silver salts 16, 17. Furthermore, some organic substances among these can also be used as stabilizers and capping agents 17. Among the different methods, the additives mentioned may influence the subsequent medical applications of AgNPs.

AgNPs are recognized for wide-spectrum and high antimicrobial activity, they can effectively kill a variety of pathogens even at very low concentrations 18, including (i) bacteria, such as *Escherichia coli*, *Klebsiella pneumonia*, *Staphylococcus aureus*; (ii) fungi, such as *Candida albicans*, *Aspergillus niger*; (iii) virus, such as Hepatitis B virus (HBV) and human immunodeficiency virus (HIV). Besides, some studies have shown that AgNPs have nematicidal and anthelmintic activity. The mainstream recognition of the antimicrobial mechanisms of AgNPs includes destructing bacterial cell walls, producing reactive oxygen species (ROS) and damaging DNA structure 18, 19. Unlike the risk of antibiotic resistance which may limit medical applications, rare AgNPs resistance of bacteria is observed 20. This may be attributed to the simultaneous multiple antibacterial mechanisms of AgNPs.

In recent years, the anticancer effect of AgNPs has been widely studied. AgNPs play an efficient role against a variety of cancer both *in vitro* and *in vivo*, including cervical cancer, breast cancer, lung cancer, hepatocellular carcinoma, nasopharyngeal carcinoma, hepatocellular carcinoma, glioblastoma, colorectal adenocarcinoma, and prostate carcinoma 21-23. The anticancer activity of AgNPs is affected by inherent properties, including size, shape and surface charge 24-26. Generally speaking, the smaller the particle size, the higher the biological activity. To obtain an ideal anticancer agent with high biological activity, our team successfully synthesized a kind of very small silver particles which reached up to Ångstrom (Å; one-tenth of a nanometer) scale and determined the stronger anticancer activities of silver Ångstrom particles (AgÅPs) compared with AgNPs 21. In addition, exposure time and dose are also considered as crucial factors. Longer exposure time and higher dosage will trigger stronger anticancer effects. Some possible mechanisms involving the anticancer effects of AgNPs have been proposed. AgNPs can cause apoptosis or necrosis by destroying the ultrastructure of cancer cells, inducing ROS production and DNA damage, inactivating enzymes, as well as regulating signaling pathways 27-29. In addition, AgNPs can also block the invasion and metastasis of tumor cells by inhibiting angiogenesis 30-32. Due to the enhanced permeability and retention (EPR) effect, tumor cells preferentially absorb NPs-sized bodies than normal tissues 33, 34. While the poor lymphatic drainage in the tumor enables nanoparticles to stay and penetrate 35. This may enhance the targeted drug delivery of AgNPs. Further studies of anticancer mechanisms of AgNPs are essential to develop economical, reliable, and broad-spectrum anticancer agents.

Besides the most studied antimicrobial and anticancer activities, AgNPs have also received attention in other cutting-edge medical applications, including wound repair, bone healing, dental material filling, vaccine adjuvants, antidiabetic agents, and bioimaging. In this review, we will also briefly introduce these biomedical applications.

Considering various products containing AgNPs, such as dressings, creams, solvents, and scaffolds, it seems necessary to assess the potential toxicity of AgNPs in cells, tissues, and organs. Generally speaking, primary exposure routes include skin contact, inhalation, ingestion, and injection 36-38. These routes will distribute AgNPs to different tissues and organs, such as skin, respiratory, circulatory, nervous, hepatobiliary, urinary and reproductive systems 36-41. The deposited AgNPs may be potentially toxic to these tissues or organs by inducing cell necrosis, apoptosis or genetic mutations 42-45. For example, AgNPs deposited in the lungs can cause pneumonia and asthma 46. AgNPs may cross the blood testis barrier (BTB) and reduce the fertility of model animals and cause teratogenicity in offspring 42. Some toxicological studies on model animals have shown that the potential toxicity of AgNPs is related to the inherent properties 25, 47. Large surface area may lead to increased silver ions (Ag^+^) released from AgNPs, which may enhance the toxicity of nanoparticles. Besides the unique properties, the potential toxicity of AgNPs is closely related to dose, concentration and exposure time 24, 48-50. Exploring the pharmacodynamics of AgNPs *in vivo* may contribute to the development of bio-friendly and safe agents.

In recent years, a considerable amount of researches involving AgNPs prove enough evidence of promising medical applications of silver nanomaterials. However, the potential toxicities of AgNPs to mammals *in vivo* and cell lines *in vitro* alert us to be cautious about its utilization. This reminds us to carry out more researches to obtain safe, bio-friendly agents containing AgNPs. This article provides a review of the applications of AgNPs and potential toxicology from an objective stance with insights toward understanding deep implications for medicine.

## Synthesis of AgNPs

**Figure 1:**
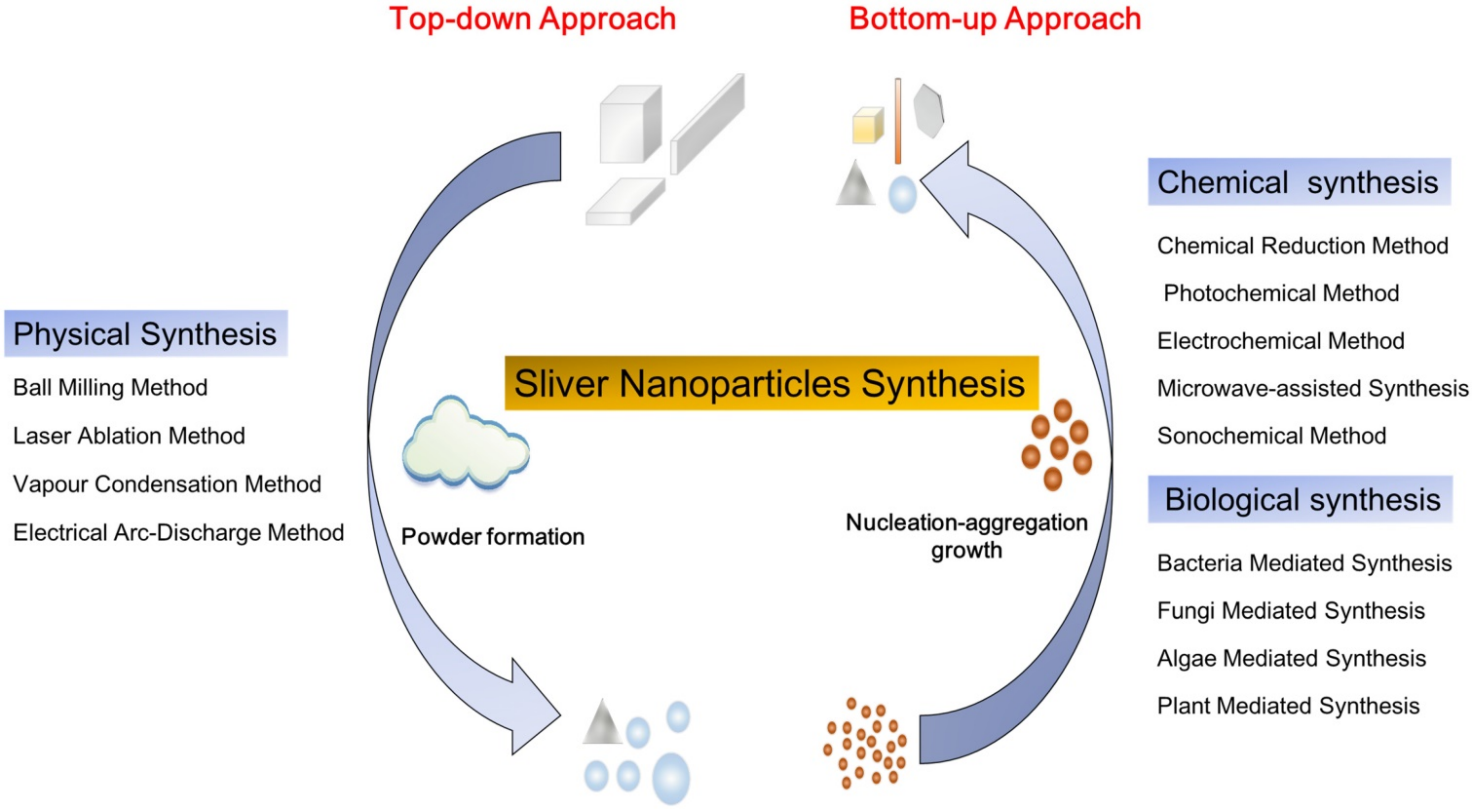
Silver nanoparticles synthesis: top-down approach and bottom-up approach, i.e. physical synthesis method, chemical and biological synthesis methods, separately. The top-down approach refers to the formation of metal nanoparticles from bulk materials, while the bottom-up approach refers to the growth of complex clusters and obtained nanoparticles from molecular components.

The synthesis methods of nanoparticles (NPs) are mainly divided into two processes: top-down and bottom-up (**Figure 1**). The top-down approach refers to the formation of metal NPs from bulk materials using various physical forces to synthesis NPs, such as mechanical energy used in ball milling, crushing and grinding; electrical energy used in the electrical arc-discharge method and laser ablation method; and thermal energy used in vapor condensation method 51. These approaches can obtain NPs between 10 and 100 nm in size. The top-down approach, mainly the physical method, may acquire pure nanoparticles without chemical additives. NPs synthesized by physical method may exhibit uniform particle size distribution and high purity. Though the physical approach does not contain chemical reagents which may harm human and environment, it brings a great challenge to prevent agglomeration due to absence of stabilizer or capping agents. Furthermore, these methods need complex equipment and external energy in the process. The bottom-up approach involves the construction of complex clusters to obtain NPs from molecular components by employing nucleation and growth processes 51, 52. The commonly used bottom-up approaches include chemical synthesis and biological synthesis, both can obtain NPs by reducing the precursor salt 52. The chemical synthesis can be coupled with alternative energies, such as photochemical 53, electrochemical 54, microwave-assisted 55 and sonochemical methods 12. Though the chemical method is carried out to quickly obtain various shapes of NPs, the use of harmful chemical additives may limit the medical applications of NPs. To overcome the shortcomings of the chemical method, the biological method has been regarded as an alternative option. The biological method usually relies on macromolecular substances in bacteria, fungi, and algae 16, such as exopolysaccharide, cellulose, and enzymes, and organic components in plant extracts such as enzymes, alcohol, flavonoids, alkaloids, quinines, terpenoids, phenolic compounds 16, 56-59. Biological synthesis is an economical, environmentally friendly, simple and reliable approach, but the components on the surface of nanoparticles must be adequately considered in the application. Based on these two approaches, frequently used methods for synthesizing AgNPs, including physical, chemical and biological methods are discussed herein.

### Physical Method

The physical synthesis of AgNPs involves mechanical processes and vapor-based processes. Energies are used to reduce particle size, including mechanical energy (ball milling method) 60, electrical energy (electrical arc-discharge method) 61, light energy (laser ablation method) 62, and thermal energy (physical vapor deposition) 6 (**Table 1**). During the ball milling progress, high-speed collisions between rigid balls, such as ceramics, flint pebbles, and stainless steels, can produce localized high pressures, which grind the metal into very fine powders 60. The electrical arc-discharge method can obtain NPs via arc discharge device under direct current (DC) power 63. The device uses the powder reagent layer as the anode and the electrodes are immersed in dielectric liquids such as hydrocarbons, liquid inert gas, and deionized water. Laser ablation method refers to the ablation of a metal plate by a high-power laser, the metal target absorbs the laser beam energy and photoions, followed by nucleation and growth of metal particles during the plasma plume cooling process and eventually synthesize NPs 62, 64. Sputtering and evaporation are two processes in physical vapor deposition. Sputtering refers to bombarding a target coating material with a high-energy electrical charge to sputter off atom or molecule that can be deposited on the substrate. While evaporation refers to heating the coating material to the boiling point in a vacuum environment and evaporating, and the evaporated material rises in the vacuum chamber and condenses on the substrate. Although physical synthesis can produce AgNPs on a large scale, AgNPs may aggregate and form large-sized particles which will affect subsequent applications. In order to avoid the re-aggregation of AgNPs, some stabilizers are used to obtain stable colloids AgNPs. For example, polyvinyl pyrrolidone (PVP) may be used as both the electrolyte and stabilizer in the synthesis of AgNPs by laser ablation method 65. Our team prepared Ångstrom silver particles, capped with fructose as stabilizer, can be stable for a long time 21. In summary, the physical method can quickly produce NPs with uniform size distribution and high purity, but complex equipment and external energy are required.

#### Ball Milling Method

Mechanical ball milling technique is to put milling balls and metal materials with a specific mass ratio as well as gas (air or inert gas) in a container rotated at a high speed. The milling time, rotating speed and the atmospheric medium in the process of ball milling are playing essential roles in the morphology of metal materials. A suitable milling time is closely related to the production of particles with a satisfactory size. The smaller size of particles, the higher surface energy, therefore particles prefer to aggregate. The temperature of the powder in the ball milling process influences the diffusivity and phase of nanoparticles 60. Generally speaking, a higher temperature of powder tends to synthesize intermetallic compounds, while lower temperature tends to obtain amorphous and nanocrystalline phases 52.

#### Electrical Arc-Discharge Method

The electrical arc-discharge apparatus consists of DC power between two silver rods, which are immersed in dielectric liquids 61, 66. During the process of arc discharge, the silver electrode is etched in the dielectric medium, and the surface of the silver electrode is vaporized because of the high temperature near the electrode. Subsequently, the silver vapor is condensed into AgNPs and suspended in the dielectric liquid. This apparatus can obtain pure AgNPs with a simple and low-cost device.

#### Laser Ablation Method

Laser ablation method refers to a pulsed laser instantaneously heat the target bulk metal immersed in water or an organic solvent to form plasma plume, followed by nucleation and growth of metal particles during the plasma plume cooling process and eventually form nanoscale clusters 62, 64. During the process of laser ablation, nanoparticles can absorb photons through multiple pathways, including plasmon excitations, interband transitions, and multiphoton absorption, which are closely related to pulse time, laser wavelength, and laser fluence. These factors, as well as the type of aqueous medium, may affect the characteristics of NPs 62. Different synthesis conditions, such as laser fluences, pulse wavelength, as well as solvent type, may affect the size of the NPs. The addition of organic stabilizers such as cetyltrimethylammonium bromide (CTAB) and PVP can enhance the dispersibility of AgNPs 11. However, it is difficult for laser ablation method to control the size distribution of NPs 62.

#### Physical Vapor Deposition Method

The basic and most commonly used physical vapor deposition processes are divided into two general categories: arc evaporation and sputtering 67. The former refers to the utilization of a cathodic arc source in a vacuum chamber or protective gases to obtain metal vapor and deposit it on a target coating material to form a thin, adherent pure metal or alloy coating. During this process, highly ionized metal vapor generates plasma 68. And the latter refers to using a high-energy electrical charge to bombard the target coating material and deposit metal on the substrate. In this process, ions and energetic atoms impact atoms and mechanically eject them from the target material. Recently, our team successfully synthesized a kind of very small silver particles which reached up to Ångstrom (Ång) scale for the first time with a self-developed evaporation-condensation system 21. A pure silver wire was fed into an explosion chamber filled with protective gas Argon, following by a high voltage of 25 kV when the wire contacted the positive electrode plate. The silver wire was exploded and evaporated to yield silver vapor plasma. Then the silver vapor was rapidly cooled and coagulated to form Ag particles in the rapid cooling chamber with a water chiller at 0-4 °C. High-intensity ultrasonic and demagnetization devices were used successively to prevent re-agglomeration of Ag particles. In conclusion, the physical vapor deposition method can obtain pure and dispersible AgNPs with small particle size, but complicated devices and external energy are required.

### Chemical Method

Chemical synthesis is currently the most common method to synthesize AgNPs (**Table 2**). The process involves the reduction of Ag^+^ (supply by silver salt precursor) to elemental silver (AgNPs) through electron transfer under certain conditions 8, 69. In general, chemical synthesis can be promoted by reducing agents such as sodium borohydride (NaBH4) and sodium citrate (TSC). The chemical method can be combined with external energy sources to prepare AgNPs, such as photochemical, electrochemical, microwave-assisted and sonochemical methods. Among these methods, the generation process of AgNPs can be divided into two parts: nucleation and growth. The monomer concentration in the solution rapid rises above the critical level of supersaturation, and triggers “burst-nucleation” and precipitation 70, 71. The precipitation of the monomer forms the nucleus, and the repetitive nucleation process promotes the continuous birth of new seeds. As the seed formation, monomer concentration drops below the critical level of supersaturation. After nucleation, the increased addition of monomer induces the growth of nuclei and forms NPs with a larger size. During the synthesis process, stabilizers such as PVP and CTAB are usually used to stabilize and disperse AgNPs. Even though the chemical method of AgNPs is a reliable, high-yield, time-saving and controllable route, it must be noted that chemicals used in this method may cause environmental pollution.

#### Chemical Reduction Method

**Figure 2:**
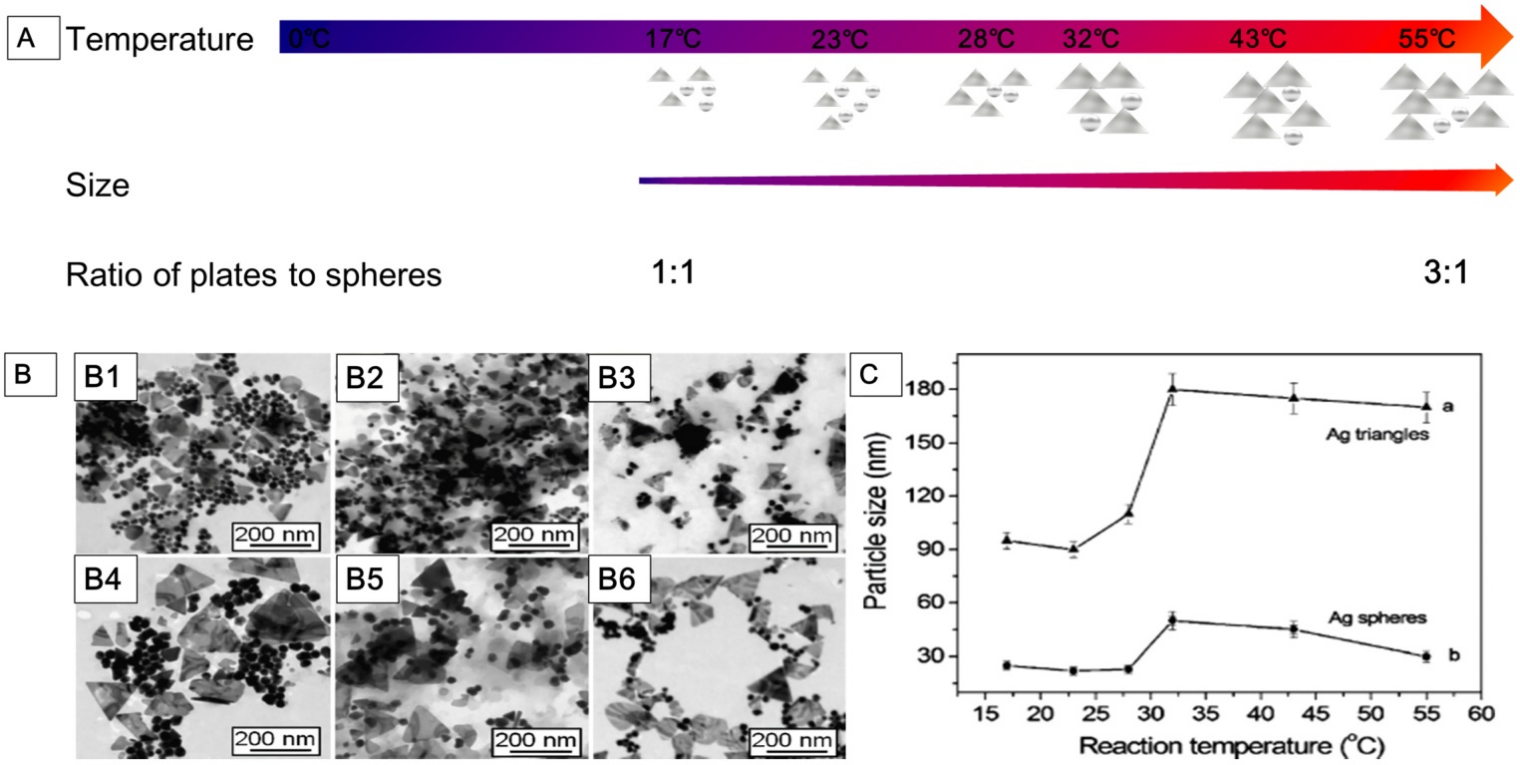
** AgNPs synthesized at various temperatures.** (**A**) The shape and size of AgNPs obtained in reaction systems at different temperatures ranged from 17 °C to 55 °C. (**B**) Transmission electron microscope (TEM) images of the AgNPs synthesized at different temperatures: (B1) 17 °C; (B2) 23 °C; (B3) 28 °C; (B4) 32 °C; (B5) 43 °C; (B6) 55 °C. (**C**) The average size of AgNPs (curve a: silver nanoplates; curve b: silver nanospheres) synthesized at different temperatures. Adapted with permission from 78, copyright 2011 Nanoscale Research Letters.

Chemical reduction is a reliable method for preparing colloidal AgNPs in organic solutions or water. AgNPs with desired shapes can be obtained by chemical reduction method, such as nanosphere, nanoprism, nanoplate, nanowire, nanocube, and nanorod. The chemical reduction method includes three components: salt precursor, reducing agent, and stabilizer. Silver precursors can be effectively reduced to AgNPs by different reducing agents with the presence of a stabilizer. There are several alternative silver precursors continuously providing monomers for nucleation, such as silver nitrate 69, silver ammonia ( ie. Tollens reagent) 72, silver sulfate 73, and silver chlorate 74. Frequently used reducing agents may affect the growth of nuclei, including NaBH4, hydrazine, N, N-dimethylformamide, TSC, ascorbic acid, ethylene glycol, polysaccharides, and formaldehyde. The types and ratio of precursors and reducers, as well as the temperature and pH of the solution, may influence the characteristics of AgNPs 75-78. The nucleation and subsequent growth of the particles in the chemical reduction process can be controlled by alternating the components and adjusting the reaction parameters. For example, Jiang et al. 78 studied the role of temperature in the synthesis of AgNPs by chemical reduction method (**Figure 2**). At the reaction temperature range of 0 to 55 °C, the low temperature significantly slowed down the generation of nuclei and growth, therefore, it took a longer time to complete the reduction of precursors. From 17 to 55 °C, the reaction rate increased with rising temperature, as well as the size of nanoparticle (**Figure 2A, 2B**). There was a size jump in the reaction synthesis at around 32 °C, i.e., the size of nanoparticles increased rapidly from around 90 nm to 180 nm for the edge length of plate AgNPs and from around 25 nm to 48 nm for the diameter of spherical AgNPs (**Figure 2C**). The ratio of the plate to spherical nanoparticles might be fundamentally dependent on the amount of single-crystal and twin structures formed at the nucleation process. In this experiment, the amount of spherical AgNPs decreased while the plate ones increased with the temperature rising. The ratio of the plate to spherical AgNPs was 1:1 at 17 °C, while 3:1 at 55 °C (**Figure 2A**).

#### Photochemical Method

The photochemical method refers to reduce the precursors to AgNPs under the illumination. The silver precursors and solution in the luminescent region produce reduced free radicals and hydrated ions, which can reduce Ag^+^ to Ag^0^ *in situ* directly. Light sources involving in the photochemical method include ultraviolet light, sunlight, and laser light, among which ultraviolet light is most commonly used. The source, intensity and wavelength of the light, and the irradiation time may affect the synthesis of AgNPs 79. For example, prolonging the irradiation time and increasing the irradiation intensity during photochemical synthesis may promote the reduction of Ag^+^ 79. The photochemical method has the unique advantage of synthesizing highly dispersible nanoparticles *in situ* in the illumination region. Therefore, the photochemical method can obtain AgNPs on the surface of various media, such as polymeric films, glass, and cells which are illuminated. The photochemical method typically requires relatively simple equipment and can be carried out at room temperature without harmful or strong reducing agents. The reactions can be terminated or attenuated by stopping the illumination.

#### Electrochemical Method

Electrochemical method can form an electric potential in the electrolyte and reduce Ag^+^ to Ag^0^ 80. The nucleation and growth of AgNPs occur almost simultaneously under the external electric field. Electrochemical method can synthesize AgNPs with different sizes by adjusting the current density. Besides, electrode types, electrolytes, and solvents are also important in the synthesis of size-controlled AgNPs. In the synthesis process, increased precursor concentration and enhanced current intensity, as well as prolonged implementation time, will obtain more AgNPs with smaller size 81. To obtain dispersed and stable AgNPs, stabilizers and capping agents are optional additives. The steric hindrance formed by these additives will prevent the aggregation of AgNPs. The electrochemical method has the advantages of easy reaction control, mild reaction conditions, and less environmental pollution.

#### Microwave-assisted Method

Microwave-assisted method refers to rapid heating the silver precursor by microwave irradiation, which may promote the generation of nuclei* in site* 82. Several factors may influence the microwave-assisted synthesis of AgNPs, including the concentration of precursor and the type of stabilizer, power input and irradiation time of the microwave, dielectric constant, refractive index of the medium and chirality of reducing agents 55. Water and alcohol are ideal media for microwave heating stabilizer because of their high dielectric losses 83. For example, polar molecules such as H_2_O attempt to orient the electric field in the microwave. When dipolar molecules attempt to reorient relative to an alternating electric field, they lose energy in the form of heat which may contribute to the reduction of Ag^+^. Microwave-assisted method has the advantages of high energy conversion efficiency, time-saving, cleanliness, and convenience, most importantly, it can be used to obtain large-scale production of high dispersive AgNPs.

#### Sonochemical Method

Sonochemical method refers to the cavitation effect generated by ultrasonic irradiation, which produces a local hot spot and promotes the synthesis of AgNPs 84. The instantaneous high pressure and microjet generated by ultrasonic irradiation can uniformly mix the solution and generate bubbles, which may suddenly collapse when the bubbles grow. The adiabatic compression of the gas phase in the bubble creates a local hot spot, which accelerates the contact of Ag^+^ with the reducing agent and rapidly reduces it to AgNPs. Ultrasound prevents the agglomeration of nanoparticles in the aqueous solution to decrease the size of AgNPs. Besides the high temperature, other factors such as pressure, pH, high-speed microjet, and high cooling rate may also contribute to the synthesis process. In summary, the sonochemical method is a simple, economical, and environment-friendly technique for preparing colloidal silver nanoparticles.

### Biological Method

In recent decades, a variety of microorganism- and plant-mediated biological syntheses of AgNPs are developed. The microorganisms can evolve metal tolerance genes and metal bioconcentration capability to survive in an extreme silver-rich environment 105, 106. These adaptive evolutionary mechanisms include altering and decreasing the cytotoxicity of metal and resulting formation of AgNPs. AgNPs can be regarded as “by-product” of the resistance mechanism of microorganisms against free Ag^+^. Plant mediated synthesis can reduce Ag^+^ to Ag^0^ using functional groups such as O-H and =C-H in organic components and their derivatives contained in the extract of plant parts 107. Commonly used plant parts include bark, peel, callus, leaves, flower, fruit, stem, seed, and rhizome. In the process of biosynthesis, various biological components act as reducing agents, such as exopolysaccharide, peptides, nitrate reductase, reducing cofactors, c-type cytochromes, separated from microorganisms, and starch, cellulose, chitin, dextran, alginates, separated from plants. However, the organic components in the biosynthesis of AgNPs require to be further studied due to their complex interaction with AgNPs and the diversity of plants. Compared with physical or chemical methods, biological method can be carried out at normal temperature and pressure and avoid the use of toxic or hazardous additives. In this part, we will introduce several microbial and plant synthesis approaches of AgNPs, as well as the mechanisms involved in these processes.

#### Bacteria-Mediated Synthesis

Since Tanja Klaus et al. firstly reported the phenomenon of aggregation of AgNPs in *Pseudomonas stutzeri* AG259 in 1999 105, series of bacteria, both Gram-negative and Gram-positive, are been screened for the synthesis of AgNPs (**Table 3**). The property of bacteria to survive in an extreme silver-rich environment might contribute to the accumulation of AgNPs 105, 108. Depending upon the location of the nanoparticles distribution, AgNPs may be synthesized intracellularly or extracellularly using biomass, supernatant, cell-free extracts, and derived components of the bacteria. Among these two modes, extracellular method is advantageous over intracellular method due to the convenience of recovery of AgNPs. The abilities and mechanisms of strains used in the biosynthesis of AgNPs are different from each other due to the organic substances. Various organic substances in bacteria can be used as reducing agents, such as exopolysaccharide, peptides, reductase, cofactors, c-type cytochromes, and silver-resistant genes. Among these, several enzymes have been involved in synthesizing AgNPs, such as nitrate reductase and lactate dehydrogenase; and peptides with special amino acid, such as methionine, cysteine, lysine, and arginine, may attach on the surface of nuclei and act as reducing agents 109. Nitrate reductase, a kind of NADH-dependent enzymes, has gained more attention in the bacteria-mediated synthesis of AgNPs. Nitrate reductase can participate in the electron transport chain, and subsequently creates a miniature reducing environment by transferring hydrogen atoms. The enzyme gains electron from NADH, oxidizes it to NAD^+^, and undergoes oxidation to reduce silver ions to AgNPs 18, 109. Some organic substances can also act as stabilizers and capping agents for AgNPs to prevent particle aggregation 18, 110. The mechanisms of bacteria-mediated synthesis of AgNPs still need to be further explored. In conclusion, bacterial-mediated synthesis of AgNPs is a simple, effective, and environmentally friendly method.

#### Fungi-Mediated Synthesis

Fungi-mediated synthesis of AgNPs is an effective and straightforward approach 111, 112. According to the location of nanoparticles, fungi-mediated synthesis can obtain intracellular and extracellular AgNPs using mycelia and fungal cell-free filtrate, respectively 113, 114 (**Table 3**). Compared with intracellular synthesis, the extracellular synthesis of AgNPs using fungi is preferred due to the advantages of convenient collection and downstream processing. Plenty of fungi, due to their unique abilities of metal bioconcentration, high tolerance in the metal-rich environment, rapid mycelial growth, various extracellular enzymes secretion, and economic viability, are selected for biosynthesis of AgNPs 115, such as *Fusarium oxysporum* 116, *Trichoderma harzianum* 57, *Penicillium polonicum* 117, *Phomopsis liquidambaris* 118. However, some fungi, such as *F. oxysporum* 111, are recognized to be potentially pathogenic, which may result in health risk in subsequent applications. While the AgNPs synthesized by extracellular method using the fungal extract can be purified by washing or precipitating unnecessary fungal components. Various organic components of fungi play an important role in the synthesis of AgNPs, such as nitrate-dependent reductase, xylanases 119, naphthoquinones and anthraquinones, and quinine derivates of the latter two, are involved in the reduction of silver precursor. In addition, some proteins secreted by fungi can be used as capping agents to form shape-controlled AgNPs 120. Various incubation conditions might influence the characteristics of AgNPs, such as the types of carbon and nitrogen sources, temperature and light source 56**.** In conclusion, fungi mediated synthesis of AgNPs is a convenient, effective, low-cost and energy-saving biological method. However, reducing potential pathogens on the surface of AgNPs should be considered to obtain safe products.

#### Algae-Mediated Synthesis

Algae, as one of the most potential coastal renewable living resources, have received more attention in the biosynthesis of nanometer materials in recent years (**Table 3**). Algae contain a variety of biologically active organic matters, such as carbohydrates, polysaccharides, enzymes, proteins, vitamins, pigments and secondary metabolites 17, 121, 122. These abundant organic compounds make algae an ideal candidate for biosynthesis of AgNPs. These active organic matters may be used as reducing agents to form size- and shape-controlled AgNPs, including spheres, triangles, cubes, rods, wires, hexagons, pentagons and wires. The roles of many algae in biosynthesis of AgNPs are verified, including *Cyanophyceae*, *Chlorophyceae*, *Phaeophyceae*, *Rhodophyceae* 123. These studies support algae as a promising bioresource for the synthesis of AgNPs with various shapes and sizes. Biomolecules in algae extracts, such as amino acids, proteins and sulfated polysaccharides, can also act as stabilizers or capping agents in the biosynthesis of AgNPs with variable properties 124. The specific factors involved in the algae-mediated synthesis of AgNPs are necessary to be identified and determined, including the ratio of silver precursor to algae extract, mixture pH, incubation time and temperature 125. In conclusion, the biosynthesis of AgNPs using algae extract provides a facile, sustainable and eco-friendly method. Various algae can be considered as candidates in the biosynthesis of AgNPs due to their unique properties of rapid growth, high metal accumulation ability and abundant organic content.

#### Plant-Mediated Synthesis

Plant-mediated synthesis of AgNPs, as a promising approach, has received great attention in recent years. Extracts from different parts of the plants, including bark, peel, callus, leaves, flower, fruit, stem, seed and rhizome, are involved in biosynthesis of AgNPs with various sizes and shapes 59 (**Table 4**). These extracts from different plant parts contain organic components such as enzymes, alcohols, flavonoids, alkaloids, quinines, oils, terpenoids and phenolic compounds 126, 127. There are different functional groups in these organic components 58, like hydroxyl, carbonyl, amidogen, which may contribute to the reduction of Ag^+^ to Ag^0^. A variety of plant extracts, including the components mentioned above and plant derivatives such as starch, cellulose, chitin, dextran and alginates, act simultaneously as reducing agents and stabilizers 128. The plant-mediated synthesis of AgNPs is influenced by different reaction parameters such as temperature, reaction time, pH and concentration of plant extracts and precursors 129, 130. The AgNPs with different size and shape can be obtained by changing the synthesis parameters 129. In summary, plant-mediated synthesis of AgNPs can be controlled by a variety of reaction conditions. In addition, different parts of plant exhibit various abilities in the synthesis of AgNPs 131. The mechanisms of plant-mediated synthesis of AgNPs need more exploration. In conclusion, the plant-mediated synthesis of AgNPs using plant extract is a promising method due to its easy availability, nontoxicity, simplicity, cost-effectiveness and high reducing potential.

## Medical Applications of AgNPs

Antimicrobial and anticancer properties of AgNPs have been widely studied. Studies have shown that AgNPs have broad-spectrum antimicrobial properties against pathogens including bacteria, fungi and viruses 19, 49. Besides, AgNPs can effectively damage or kill nematodes 152 and worms 153. A variety of factors affect the antimicrobial activities of AgNPs, including size, shape, dose and stabilizer of AgNPs 49, 154, 155. Interestingly, AgNPs may have different antibacterial effects against Gram-positive and Gram-negative bacteria 156. AgNPs exhibit broad-spectrum anticancer properties. Anticancer activity of AgNPs is also affected by a variety of factors, including size, shape, dose, and exposure time 22, 157, 158. It is also realized that the surface charge of AgNPs is a potential factor. Although current specific mechanisms of antimicrobial and anticancer properties of AgNPs are still unclear, many studies have carried out hypothesis. AgNPs can inhibit the growth of bacteria or kill them by inducing membrane destruction, ROS generation, DNA damage, enzyme inactivation and protein denaturation 4, 56, 159. However, the anticancer mechanisms of AgNPs are much more complicated. Until now, it has been approved that AgNPs can inhibit the growth of tumor cells by destroying the cellular ultrastructures, inducing ROS production and DNA damage 21-23, 160. In addition, AgNPs can induce tumor cell apoptosis through inactivating proteins and regulating signaling pathways, or blocking tumor cell metastasis by inhibiting angiogenesis within lesion 31, 161. Besides antimicrobial and anticancer properties, AgNPs can also be used in other medical applications, such as bone repair 162 and wounding repair 163. And AgNPs can be regarded as an additive in dental materials or an adjuvant in vaccine. In this part, we will discuss the antimicrobial and anticancer properties and possible mechanisms of AgNPs, as well as other promising medical applications.

### Antimicrobial Application of AgNPs

#### Antibacterial Properties of AgNPs

AgNPs have been proven to effectively inhibit various pathogenic bacteria, fungi and viruses, including *Staphylococcus aureus* 164, *Escherichia coli* 165, *Pseudomonas aeruginosa* 166, *dermatophyte* 167, HIV-1, etc. 168, 169. The antibacterial effect of AgNPs against various strains of bacteria is different 156. Rather than Gram-positive bacteria, AgNPs show a stronger effect on the Gram-negative ones. This may be due to the different thickness of cell wall between two kinds of bacteria 170. Besides the bacteria strains, AgNPs may also exhibit size-, shape-, concentration-, time-, and charge-dependent antibacterial activity. In general, as particle size decreases, the antibacterial effect of AgNPs increases significantly 171. Especially when the size is less than 10 nm, AgNPs show better antibacterial activity 172. The antibacterial effect can be significantly enhanced by prolonging the treatment time of AgNPs 173. The increased bacterial mortality may be ascribed to the accumulation of AgNPs and silver ions during the exposure period. Besides, the shape of AgNPs may also influence the antibacterial activity 171, 174. By comparing the antibacterial activity of spherical, triangular, linear and cubic AgNPs, it is observed that spherical shaped AgNPs exhibit superior antibacterial effect. This phenomenon suggests that AgNPs with larger surface to volume ratio, which relates to both higher effective contact and larger reaction surface, may show stronger antibacterial activity 174. In addition, the antimicrobial activity of AgNPs is also affected by the surface charge 156, 175. Due to the presence of lipopolysaccharide, peptidoglycan and multiple groups, including carboxyl, amino and phosphate groups, bacterial membranes are primarily loaded with negative charges 170, 176. Positive charge can facilitate the adherence of AgNPs on bacterial membranes through electrostatic attraction 156. Therefore, adjusting the surface charges of AgNPs may contribute to the enhanced antibacterial effect 175. The stabilizers may influence the size, dispersion, and surface charge of AgNPs, which may involve in the antibacterial effect of AgNPs 154, 177. Some stabilizers, such as citrates, PVP 154 and polyvinylalcohol 177, have been shown to influence the bacterial effect by adjusting the characteristics of AgNPs.

Although AgNPs exhibit good antibacterial activity, the specific mechanisms have not been completely clarified. Many hypotheses have been proposed to explain the antibacterial mechanisms of AgNPs, including i) Destructing the bacterial membrane and leaking cellular contents; ii) Generating ROS and disabling the respiratory chains; iii) Destructing the DNA structure and blocking the DNA replication; iv) Inactivating enzymes and denaturing proteins. Due to these mechanisms, AgNPs exhibit broad-spectrum and effective antibacterial properties. These make AgNPs an alternative for the implementation of novel biomedical strategies, such as catheter modification, dental application, wound healing and bone healing.

#### Antifungal and Antiviral Activities of AgNPs

Some studies confirm that AgNPs exhibit good antifungal properties against *Colletotrichum coccodes*, *Monilinia sp.* 178, *Candida spp.* 179 and various plant pathogenic fungi in size- and dose-dependent manners 180. Some studies also point out that the type of culture media used in their experiments may also affect the inhibition activity 180. Besides, AgNPs also show good antiviral activity against hepatitis B virus (HBV) 181, human parainfluenza virus (HPIV) 182, herpes simplex virus (HSV) 183 and influenza A (H1N1) virus 184. AgNPs with less than 10 nm size exhibit good antiviral activity 185, 186, which may be due to their large reaction area and strong adhesion to the virus surface.

For example, AgNPs can bind to the glycoprotein knobs and inhibit the reverse transcriptase (RT) of HIV-1 and interact with the virus in size- and dose-dependent manner 169, 185. To develop AgNPs for antimicrobial applications, the detailed mechanism needs to be further studied.

#### Antimicrobial Mechanisms of AgNPs

**Figure 3:**
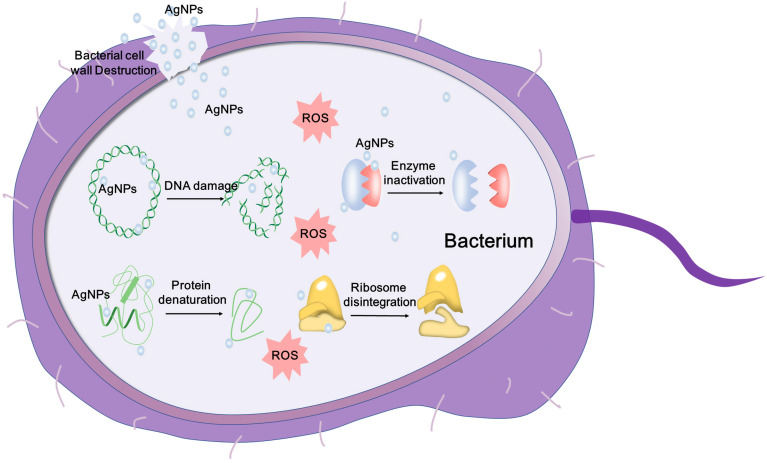
Schematic representation of the mechanisms of AgNPs against bacteria, depicting ROS-dependent pathway, DNA damage, protein denaturation and enzyme inactivation for antibacterial action of AgNPs.

The antimicrobial effect of AgNPs has been widely studied, and the mechanisms are being explored. It is observed that AgNPs can anchor and then penetrate the bacterial membrane, and subsequently trigger the destruction of cell membrane and leakage of content 187. Besides, AgNPs can influence crucial intracellular activities, such as attacking the respiratory chain, disturbing DNA replication and inhibiting cell division 188. The antibacterial mechanisms of AgNPs are illustrated in **Figure 3**. AgNPs also have a good microbicidal effect in drug-resistant fungi via influencing the cellular targets, which are involved in the drug resistance and pathogenicity. For example, Venkatraman et al. 189 demonstrated that AgNPs could affect drug sensitivities by acting on multiple cellular targets of *Candida albicans*, including fatty acids like oleic acid, which were important in the hyphal morphogenesis involved in the pathogenicity. Some studies speculate that AgNPs can saturate and adhere to the fungal hypha and eventually inactivate the fungus 180. The antiviral mechanism of AgNPs has also been deeply explored. AgNPs can be used to prevent viral infection against several virus by blocking virus contact with cells and entry steps, or directly inactivating the virus, including herpes simplex virus (HSV), human parainfluenza virus 3, vaccinia virus, chikungunya virus and respiratory syncytial virus 182, 190-192. These studies indicate that AgNPs can be used as a novel promising virucide agent. In order to develop safe and effective antimicrobial agents, the yet-to-be-determined mechanisms of antimicrobial properties of AgNPs are needed to be further studied.

#### Nematicidal and Anthelmintic Activity

Worm infection via contact with contaminated soil is one of the most common diseases among children from middle and low-income countries 193. Worm infections often lead to stunted growth, malnutrition and lagging academic performance 193, 194. According to recent studies, AgNPs may become a candidate as a novel insecticide. Saha et al. 195 confirmed that AgNPs were effective in killing filaria and larvae. AgNPs induced the cell apoptosis and destroyed parasites mainly through the generation of ROS. It was worth noting that the carbohydrate polymer not only participated in the synthesis of AgNPs, but also enhanced the filaricide activity of AgNPs. This suggested that AgNPs may be a potential preparation for filariasis control. In addition, they also tried to use AgNPs synthesized by *Acacia auriculiformis* to kill filaria, and also achieved impressive results 196. Tomar et al. 197 realized the biologically synthesized AgNPs might inhibit both egg hatch and adult motility in dose-dependent manner. That was, a higher dose of AgNPs might exhibit better anthelmintic activity. Shabad et al. 198 confirmed the AgNPs synthesized by* Ziziphus jujuba* leaf extract showed ideal ovicidal and anthelmintic activity against *Haemonchus contortus* via nutrient depletion. The combination of AgNPs and organic components separated from plants can produce a synergistic effect which may enhance anthelmintic activity. Mamun et al. 199 speculated that organic substances in *M. charantia* fruit extracts, such as glycosides, alkaloids, reducing sugars and free acids, can help biosynthetic AgNPs to protect against parasitic infections. The phytochemicals might exert effect by adhering to the gastrointestinal tract or parasite cuticles. AgNPs exhibited larvicidal activities against larvae of Anopheles stephensi and Culex quinquefasciatus, thus contributed to the prevention of malaria and filariasis 200. In conclusion, AgNPs may be used as an effective insecticidal agent to kill eggs, larvae and adult parasites. However, the mechanisms still need to be further explored.

### Anticancer Application of AgNPs

#### Anticancer Properties of AgNPs

Cancer is currently considered an important factor in morbidity and mortality worldwide 201. About 14 million new cancer cases are predicted by 2035, which will lead to a substantial impact on the economy and society around the world 202. Therefore, there is an urgent need to develop effective and advanced treatment methods to reduce the adverse effects of cancer incidence. Common treatments of cancer or tumor include surgery, chemotherapy and radiotherapy. However, side effects and limitations of conventional treatments influence the outcomes. For example, standard chemotherapy may cause serious side effects, including local reactions, such as thrombophlebitis and tissue necrosis, and systemic reactions, including myelosuppression, dysfunction of liver and kidney and immunosuppression 203. In addition, malignant tumors can develop multi-drug resistance (MDR), which may lead to chemotherapy failure 204. Therefore, it is essential to develop novel drugs to improve the therapeutic effects. In recent years, nanoparticles have attracted more attention in cancer therapeutics due to their special physical and chemical properties, which gives rise to a new field of anticancer—cancer nanomedicine 205, 206. Compared to traditional anticancer agents, metallic nanoparticles (MNPs) can be used as novel therapeutic agents or drug carriers in combination with drug candidates, and undesirable side-effects can be prevented by providing a targeted approach 207. Among these nanoparticles, AgNPs represent an ideal one in the search for anticancer or antitumor therapeutic agents 207.

AgNPs have been observed to exhibit good anticancer activities in breast cancer 158, cervical cancer 208, colon cancer 209, ovarian cancer 210, pancreatic ductal adenocarcinoma 211, lung cancer 212, hepatocellular carcinoma 213, melanoma 214, osteosarcoma 215, etc. (**Table 5**). Several studies confirm that the anticancer activities of AgNPs with various sizes, shapes and doses/concentrations are discrepant in different cancer cells 210-212, 215. In addition, other factors, such as pH of lesions, exposure time, cell lines and tumor microenvironment, also influence the anticancer activity of AgNPs 210, 211, 214. Generally speaking, AgNPs exhibit wide spectrum anticancer activity in size-, dose-/concentration- and time-dependent manners. AgNPs with smaller size can elicit enhanced endocytosis, and induce more significant cytotoxicity and genotoxicity. Compared with other shapes, spherical AgNPs exhibit better cytotoxicity due to the higher surface-to-volume ratio 216. And higher dose of AgNPs usually leads to more apoptosis than lower one. In this section, we highlight these factors.

##### Size- and Shape-dependent Manners

Nanoparticles motility in capillaries, as well as endocytosis and metabolism in tumor cells, are significantly affected by the size of AgNPs 217, 218. It has been found that the kinetics of uptake, intracellular accumulation and excretion, and the resulting cytotoxicity and genotoxicity, varied with the different sized AgNPs. In general, smaller AgNPs have higher endocytosis and exocytosis efficiency, therefore are supposed to produce greater cytotoxicity than larger particles 49, 217. To investigate the effect of nanoparticle size on distribution within tumor, Gavin Fullstone et al. 219 simulated the transport of nanoparticles in blood flow using an agent-based approach, testing the ability of 10 nm, 20 nm, 50 nm, 70 nm, 80 nm, 100 nm and 160 nm nanoparticles to traverse fenestrations with pore size of normal blood vessels and tumor-associated blood vessels.

Although 50 nm, 70 nm and 80 nm nanoparticles can effectively penetrate both, 100 nm nanoparticles cannot penetrate normal fenestrations, suggesting that there might be an optimal size for effective leakage of nanoparticles from the microvasculature into the tissue. Rona et al. 41 demonstrated that size of AgNPs could influence cellular uptake and toxicity. Smaller particles (10 nm, 20 nm) easily penetrate LoVo cells and then significantly increase intracellular ROS levels, while larger particles (100 nm) appeared mainly on the cell surface. Alicia et al. 220 also found that smaller AgNPs were more cytotoxic than larger AgNPs when studying the therapeutic effects of AgNPs on human hepatoma and leukemia. Our team 21 used an evaporation-condensation system to obtain silver particles approaching the Ångstrom dimension. By comparing AgNPs with larger size, we found Ångstrom-scale silver particles had greater cytotoxicity to tumor cells, but did not induce notable toxicity on normal tissues.

The applications of AgNPs can be extended by tailoring the shape of nanoparticle, which may optimize the physicochemical and biological properties of AgNPs 26, 221. The shape-controlled AgNPs can be obtained by changing the parameters in different synthesis methods. Though AgNPs with various shapes are prepared, such as sphere, triangle, cuboid, rod, tube, disk and wire, only a few among these are chosen for anticancer therapy. The cellular uptakes of AgNPs, as well as particle-to-cell or particle-to-protein interactions, are partly dependent on the shape of nanoparticles 216, 222. In general, spherical AgNPs may display stronger endocytosis and more active anticancer effect than other shapes. Because it is more efficient for spherical AgNPs than non-spherical nanoparticles to pass through vascular endothelium, and their higher specific surface area is more beneficial for them to enter cancer cells 216, 222. In addition, the active or weak endocytosis may be related to the different membrane bending energies of various shaped AgNPs. Ying Li et al. 223 compared the internalization rates of spherical-, cubic-, disk- and rod-shaped nanoparticles to find out the shape effect on endocytosis. They realized that the spherical nanoparticles exhibited the fastest internalization rate, followed by the cubic nanoparticles, while the disk- and rod-shaped nanoparticles exhibited the slowest internalization rate. After analyzing the free energies of four shaped nanoparticles, they speculated that the membrane bending energy of nanoparticles during endocytosis might be the main factor inducing the shape effect of the nanoparticles. Among these four shaped nanoparticles, compared with the non-spherical, the spherical nanoparticles only needed to overcome a minimal membrane bending energy barrier, while the disk shaped nanoparticles faced a larger free energy barrier caused by stronger membrane deformation. In order to understand the effect of more complex shaped particles on cellular uptake, Yuanzu He et al. 224 treated LnCAP cells with particles of different keyboard character shapes and compared the cell endocytosis. Compared with shapes without sharp features, like number 0, letter O and pound key, the rod-like microparticles, such as number 1, letter I, and arrow key, were more likely to adhere, penetrate and enter the cancer cells. The results explained that the shapes of microparticles with sharper angular features and higher aspect ratio might have a higher chance to contact and be internalized by cancer cells.

##### Dose and Exposure Time

The AgNPs exhibit dose- and time-dependent cytotoxicity against cancer 21, 225-227. In general, increased dose and prolonged exposure time can cause more tumor cell apoptosis 228, 229. Increasing dosage and prolonged exposure time can provide more opportunities for AgNPs to enter cells and trigger multiple anticancer mechanisms. Muthu et al. 226 studied the anticancer effect of AgNPs on Dalton's lymphoma ascites (DLA) cell lines and found that AgNPs showed dose-dependent cytotoxicity to DLA cells through activation of caspase 3 enzyme, ultimately inducing apoptosis. Bita Mousavi et al. 230 found that AgNPs synthesized by *Artemisia turcomanica* leaf extract showed both dose- and time-dependent anticancer effect on gastric cancer cell line. Although increased dose of AgNPs and prolonged exposure time can result in better anticancer effects, the potential toxicity to normal tissues needs to be carefully considered.

##### Surface Charge and Protein Corona

Surface charges participate in the formation of AgNPs surface chemistry, which play an important role in cytotoxicity 231-233. The surface charges of AgNPs determine the binding with serum albumin, as well as the adhesion and uptake of cells 25. Negatively charged and neutrally charged AgNPs can adhere to cell membranes but internalize in small amounts, while positively charged AgNPs exhibit more efficient cell membrane penetration and internalization 25. Besides, the positively charged AgNPs tend to stagnate on the surface of the tissue and the lumen of the blood vessels for a long time, which may be beneficial for the targeted delivery of anticancer agents 234. AgNPs with opposite surface charges exhibit different cytotoxicity in tumor cells. The greater cytotoxicity and more ROS production are observed in tumor cells exposed to high positive charged AgNPs 234. Nanoparticles exposed to a protein-containing medium are covered with a layer of mixed protein called protein corona 235. The electrostatic interactions between proteins and nanoparticles contribute to the formation of protein corona 236. Some proteins may undergo conformational changes during the formation of protein corona 235. Protein corona has an important effect on the absorption, accumulation and subsequent behaviors of nanoparticles in cells 237. It is proved that AgNPs with protein coronas enter cells via receptor-mediated endocytosis and subsequently induce mitochondrial dysfunction and cell apoptosis 238. By comparing nanoparticles without protein coronas, it is realized that the formation of protein coronas around AgNPs can be a prerequisite for their cytotoxicity.

#### Anticancer Mechanisms

**Figure 4:**
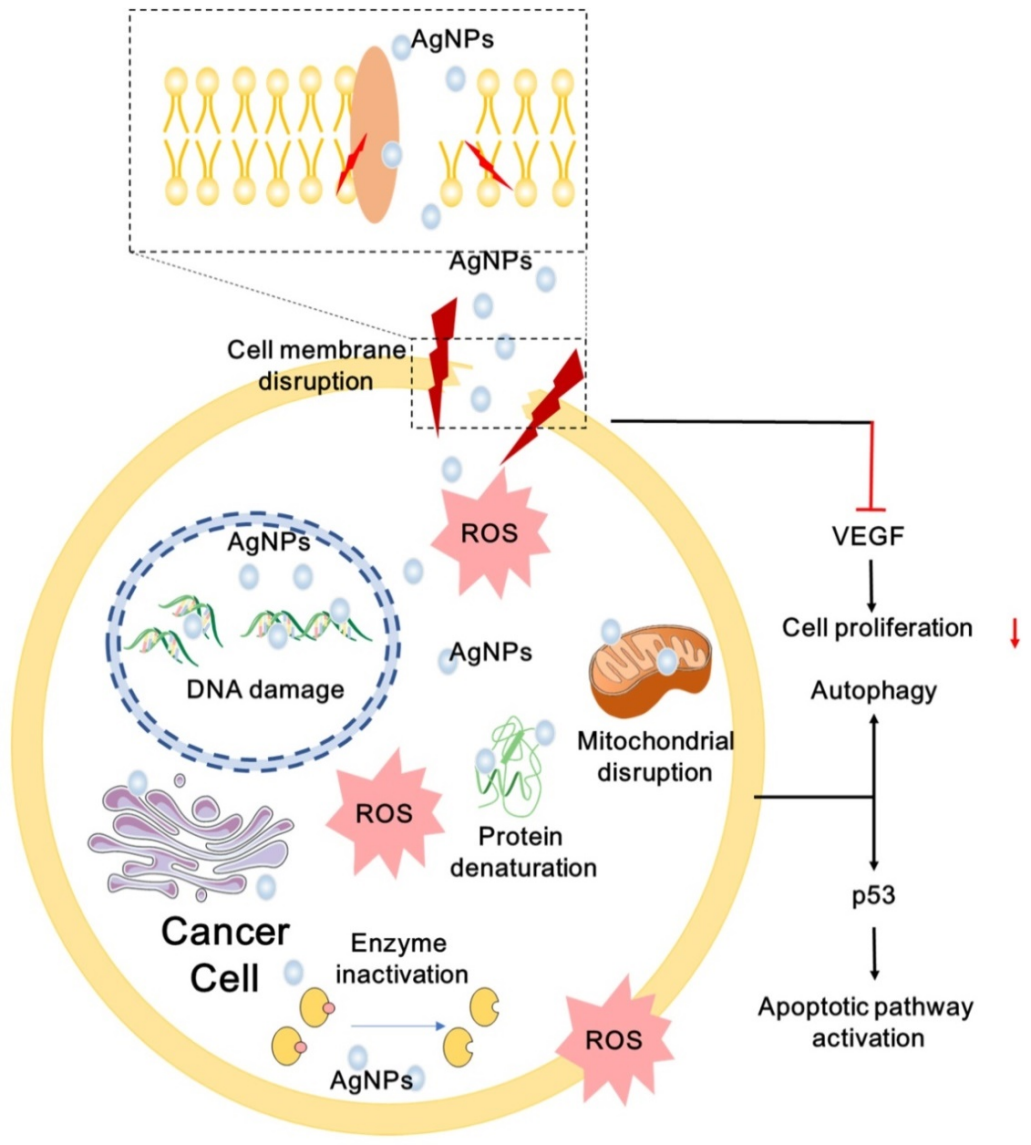
A schematic diagram of anticancer mechanisms of AgNPs. AgNPs can destroy the ultrastructure of cancer cell, induce ROS generation and DNA damage, promote apoptosis and autophagy by regulating multiple signaling pathways.

AgNPs have broad-spectrum anticancer activity via multiple mechanisms 21, 239, 240. Numerous experiments *in vitro* and *in vivo* have proved that AgNPs can decrease the proliferation and viability of cancer cells. AgNPs can cause apoptosis and necrosis by destroying the ultrastructure of cancer cells, inducing the production of ROS and DNA damage 21, 241. AgNPs can promote apoptosis by up- or down-regulating expression of key genes, such as p53 242, and regulating essential signaling pathways, such as hypoxia-inducible factor (HIF) pathway 243. Cancer cells treated with AgNPs may also show cell cycle arrest 160, 244. Several cancer cells exposed to AgNPs undergo sub-G1 arrest and apoptosis. Besides, AgNPs can also reduce distant metastasis by inhibiting tumor cell migration and angiogenesis 28, 245. Multiple anticancer mechanisms of AgNPs are described in **Figure 4**. In order to develop safe and effective anticancer agent, more mechanisms for anti-cancer effects of AgNPs remain to be explored. Here, we summarize the possible anticancer mechanisms of AgNPs both* in vitro* and* in vivo*.

##### Ultrastructural Destructions of Cancer Cells

Destruction of ultrastructures such as cell membranes and intracellular organelles leads to cell apoptosis and necrosis 21. Tumor cells exhibit intact cell structure under light microscope, such as round nuclei, intact nuclear membrane, homogeneous chromatin, normal mitochondria and rough endoplasmic reticulum 40. The ultrastructural changes of AgNPs-exposed tumor cells are in a dose- and time-dependent manner 246. Generally, the higher the concentration of AgNPs and the longer the exposure time, the more serious the damage of cell ultrastructure. TEM observation showed that AgNPs-exposed cells are suffering morphological change or cytoplasmic organelle damage, and undergoing different death patterns: apoptosis, necrosis and autophagy 40. Autophagosomes associated with apoptosis and necrosis are formed in the cytoplasm of AgNPs-treated tumor cells 247. AgNPs promote autophagosome formation through the PtdIns3K pathway, and induce autophagy in tumor cells without inhibiting lysosomal function 22. Structural and functional disruption of the actin cytoskeleton may be the cause of morphological deterioration of tumor cells exposed to AgNPs, and may be involved in inhibiting migration and invasion of tumor cells 248. Free Ag^+^ released from AgNPs is involved in the destruction of cellular membranes. Ag^+^ released by AgNPs induces oxidation of glutathione, and increases lipid peroxidation in cellular membranes, resulting in cytoplasmic constituents leaking from damaged cells 249. Our team found time-dependent morphological changes in cancer cells treated with F-AgÅPs, such as organelle compaction, nuclear fragmentation and cell blebbing 21. Tumor cells exposed to AgNPs lose their typical shape due to pseudopod contraction, decreased cell adhesion and reduced cell density. Scanning electron microscopy analysis of AgNPs-treated tumor cells reveal spherical appearance, foamed membrane and shorten filopodia 248. Tumor cells exposed to AgNPs show apoptotic cell characteristics such as loss of intact membrane, decreased contact with adjacent cells, condensed and detached from the culture plate 250.

##### ROS Production

ROS are by-products of biological aerobic metabolism, including oxygen ions, peroxides and oxygenated free radicals 251. ROS are highly active due to the presence of unpaired free electrons. ROS are controlled at a low level by normal cellular antioxidant defense mechanisms and do not affect the normal physiological activities of the cells. However, excessive ROS can produce oxidative stress that reduces the activity of biological macromolecules and damages subcellular organelles and DNA structures 252, 253. Oxidative stress trigger lipid peroxidation, impaired mitochondrial function, amino acid oxidation in proteins, enzyme inactivation and DNA/RNA damage 233, which may lead to autophagy, apoptosis and necrosis of cancer cells. AgNPs distributed in tumor cells via endocytosis can result in autophagy and apoptosis through a variety of ROS-mediated stress responses. In addition, AgNPs-induced formation of ROS may affect cellular signal transduction pathways, which may participate in the activation of apoptosis 254. For example, the mitochondrial function can be inhibited by AgNPs via disrupting mitochondrial respiratory chain, suppressing ATP production. Besides, ROS induced by AgNPs may ultimately lead to DNA damage 255. Superoxide radicals directed to mitochondria may enhance mitochondrial outer membrane permeabilization (MOMP) and the release of Cyt *c*, destroy the electron transport chain, and impair mitochondrial function 256. Some factors influence the generation of ROS induced by AgNPs. Smaller size and higher concentration of AgNPs exhibit higher induction of ROS and stronger cytotoxicity, and sharp increased ROS appear in different cancer cells treated with AgNPs 220.

##### DNA Damage

AgNPs can induce ROS production to disrupt DNA structure, or directly contact with DNA to cause DNA mutations 209, 241, 248. High levels of ROS can generate damage to DNA double helix in a concentration-dependent manner, including breaking the single or double-stranded DNA, affecting base modifications and DNA cross-links 241, 253, 257. AgNPs-treated cancer cells may exhibit DNA methylation, DNA base pairing errors, DNA repair defects and increased chromosomal aberrations 209, 248, 258. AgNPs may play an important role in the regulation of gene expression of cells. AgNPs inhibit the proliferation of cells and trigger DNA repair defects by down-regulating the functions of proteins involved in cell cycle progression and DNA repair 259. For example, proliferating cell nuclear antigen (PCNA) gene plays an important role in DNA synthesis and repair as a cofactor for DNA polymerase. PCNA is down-regulated in AgNPs-exposed cells. While the up-regulation of the apoptotic precursor protein Bax suggests that AgNPs initiate apoptosis via the mitochondrial pathway 260. AgNPs-treated cells may undergo S phase, G2/M phase and sub-G1 cell cycle arrests in a concentration-dependent manner, as well as the increased number of G0/G1 phase cells, which may be prone to apoptosis 244, 258, 261. AgNPs can not only induce apoptosis through ROS-mediated DNA damage, but also destroy DNA structure directly via Ag^0^ and Ag^+^ released by AgNPs 157. The DNA double helix structure is composed of four bases of adenine, guanine, cytosine and thymine by strictly complementary base pairing. Base pairs are bounded by hydrogen bonds to form a unit of DNA double helix. The destruction of hydrogen bonds decreases the stability of DNA structure. Tsuneo Ishida 157 analyzed the activities of AgNPs in the nucleus. Silver could form a complex containing silver within DNA. Ag^+^ caused DNA damage by replacing the hydrogen bonds in the G≡C and A=T base pairs. The Ag atom was twofold coordinated by two N atoms to form N-Ag^+^-N complex in G≡C base pair, and other complex structures appearing in the base pair were O-Ag^+^-N (G≡C base pair), N-Ag^+^-O (both G≡C and A=T base pairs). DNA damage caused by these complexes might be a factor in triggering cancer cell apoptosis.

Generally speaking, AgNPs can exert anti-cancer effects through multiple pathways. Bandyopadhyay et al. 262 confirmed that AgNPs could exhibit antitumor properties through multiple channels, including triggering cell morphological changes, ROS generation, and nuclear fragmentation, while exhibited minimum toxicity in normal peripheral blood lymphocytes. The considerable anticancer activity and histocompatibility might relate to the types of reducing agent and stabilizer.

##### Inactivate Proteins and Regulate Signaling Pathways

In the development and progression of tumors, many signaling pathways are involved in controlling cell growth and proliferation, apoptosis and viability, and can participate in more complex signaling networks that contribute to tumor progression, such as tumor microenvironment (TME), angiogenesis and inflammation 263. Some proteases and cytokines are also involved in these regulations, such as vascular endothelial growth factor (VEGF), matrix metalloproteinase (MMPs) and fibroblast growth factor 2 (FGF-2), etc. AgNPs have been confirmed to inhibit tumor proliferation, invasion and angiogenesis by regulating the associated signaling pathways or inactivating proteins. For example, AgNPs can regulate the HIF signaling pathway 161. In general, rapid proliferation of tumor cells and irregular vasculature cause hypoxic TME 264-266. HIF-1 level is up-regulated in hypoxic TME, followed by activation of target genes that in response to hypoxia. These genes contribute to cellular activities, such as cell proliferation, angiogenesis, and eventually lead to failure of cancer treatment 161. Therefore, HIF-1 is a potential target for cancer treatment. It has been demonstrated that hypoxia can weaken HIF-1α-mediated autophagy 247. Tieshan Yang et al. found that AgNPs could disrupt the HIF signaling pathway by attenuating HIF-1 protein accumulation and downstream target genes expression 161. AgNPs can also inhibit the progression of tumors by inhibiting MMPs activity. MMPs are known as protein family and classified into different evolutionary groups according to their primary sequences 267. MMPs play a dominant role in tumor progressions, such as tumor cell proliferation, invasiveness and distant metastasis, evasion of immune surveillance, and angiogenesis 267, 268. Therefore, MMPs are considered as potential targets for cancer therapy 31. In order to obtain antitumor drugs with targeting capabilities, some teams have attempted to develop inhibitors against members of MMPs.

Other signaling pathways and proteases involved in tumor progression have also been highlighted. Melissa M Kemp et al. 245 found that AgNPs could effectively inhibit FGF-2-induced angiogenesis. Their results suggested that AgNPs may have great potential for inhibiting pathological angiogenesis in cancer. Eom et al. 27 indicated that AgNPs induced cytotoxicity, including DNA damage, cell cycle arrest and apoptosis, by activating the p38 MAPK signaling cascades. These studies may inspire the development of anticancer agents containing AgNPs. In view of the complex signaling pathways and various proteins involved in the regulation of tumor development and progression, anticancer mechanisms of AgNPs by regulating intracellular signaling pathways and inactivating proteins still need to be further explored.

##### Inhibit Migration and Angiogenesis

Numerous studies have confirmed that AgNPs can inhibit migration and invasion of tumor cells in concentration- and dose-dependent manners 23, 30, 32, 269. Migration and invasion are important hallmarks of cancer progression and deterioration 270. Although it has been observed that AgNPs can inhibit tumor invasion 269, the specific mechanism is still unclear. It is hypothesized that AgNPs may decrease the protein expression of cytokines and growth factors within cancer cells, or reduce the enzymatic activity of MMPs. VEGF is an important signaling protein involved in vasculogenesis and angiogenesis, which plays a crucial role in tumor growth and metastasis 32. Various studies support that AgNPs can deprive cancer cells of both nutrients and oxygen via inhibiting angiogenesis. It has been demonstrated that AgNPs can inhibit VEGF-induced angiogenesis by inactivating PI3K/AKT pathway 271. Besides, AgNPs can block VEGF-induced Akt phosphorylation, this may influence the proliferation and migration of cells 272. Another study has proved that AgNPs can disrupt the HIF-1 signaling pathway, thus lead to inhibition of angiogenesis 161.

### Other Medical Applications

The special physicochemical properties of AgNPs make the nanoparticles and composites having considerable application prospects in the biomedical field. Besides the antimicrobial and anticancer applications mentioned above, AgNPs exhibit good properties in promoting wound repair and bone healing, as well as inhibition of inflammation. AgNPs can also be used as an additive in dental materials and adjuvant in vaccines.

#### Wound Repair

The wound healing is closely related to the prognosis of surgical treatment. The rapid development of nanotechnology in recent years has provided a new therapeutic strategy for healing wounds, but the specific mechanisms of AgNPs on wound healing still need more exploration. Jun Tian et al. 288 found that AgNPs could increase wound healing rate with less hypertrophic scarring in the thermal injury model. Compared with the healing time of deep partial-thickness wounds treated with silver sulfadiazine, the AgNPs treated group could heal in a shorter period and a superior cosmetic appearance was observed, including nearly normal hair growth and less hypertrophic scarring. In the healing process, lower level of TGF-β and increased level of interferon-γ were detected at the same time in AgNPs treated group, while the former was related to keloids and hypertrophic scars, and the later was involved in the inhibition of fibroblast proliferation. In addition, higher level of VEGF mRNA detected in keratinocytes at the edge of the wound suggested that AgNPs might promote wound healing by inducing angiogenesis. These results indicated that AgNPs could participate in wound healing by regulating various cytokines and achieve cosmetic effects. Other mechanisms of AgNPs in wound repair are being explored. AgNPs can remain in the cytoplasm of fibroblasts in skin biopsies, and promote the reconstruction of dermis and epidermis 289. Some studies prove that AgNPs can induce the proliferation and migration of keratinocytes, decrease the amounts of collagen and hydroxyproline, and promote the differentiation of fibroblasts into myofibroblasts, which may help wound early adhesion, contraction and closure 290. Besides, AgNPs can promote wound healing by regulating the production of cytokines or proteins, such as inflammatory cytokines, VEGF and MMPs 163, 291. The above studies of AgNPs on wound repair broaden our understanding of the activity of AgNPs in cellular events. The role of AgNPs in wound repair is positive for clinical wound care and postoperative results.

#### Bone Healing

Bone is an active tissue with regenerative and restorative capabilities. The self-repairing capability of bone is usually compromised when bacterial infection occurs in bone defects. Bone grafts are commonly implanted to replace or restore large defects that usually resulted from severe trauma, tumor resection or genetic malformation. Orthopedic infections are usually related to bone destruction and implant loose 292. AgNPs can be used as doping materials for synthetic bone scaffolds. AgNPs-implanted crystallized hydroxyapatite (HA) or titanium scaffolds display strong antibacterial ability against both Gram-positive and Gram-negative bacterial strains 162. AgNPs can promote fracture healing as an osteoconductive biomaterial. For example, AgNPs can naturally stimulate the osteogenic differentiation and matrix mineralization of MC3T3-1 cells 293. In a mouse model, AgNPs has been proved to stimulate proliferation and osteogenic differentiation of mesenchymal stem cells (MSCs)* in vitro*, and promote the healing process of bone fracture 294.

#### Dental Applications

Plaque biofilm formation is one of the causes of dental diseases. AgNPs have been incorporated into some dental biomaterials for reducing biofilm formation due to its antibacterial activity. Polymethyl methacrylate (PMMA), also known as acrylic resins, and composite resins are applied for the fabrication of dentures, but potential harmful organisms are likely to adhere to the rough surface of dentures 155. AgNPs incorporated in PMMA can improve the antibacterial effect of dental material. It is proved that PMMA-AgNPs showed great anti-bacterial effect by continuous releasing of Ag^+^ even in 28 days. It is highlighted that increased loading of AgNPs in PMMA also improved the mechanical properties 155. While Acosta-Torres et al. demonstrated PMM-AgNPs could efficiently decrease the adherence of Candida albicans and exhibit no obvious genotoxicity or cytotoxicity. Comparison study of the anti-bacterial and anti-biofilm efficacies of AgNPs capped with carboxymethyl cellulose and sodium alginate, respectively, showed that carboxymethyl cellulose-capped AgNPs exhibited stronger inhibition to Gram-negative organisms, which were primarily responsible for periodontal infection 295.

#### Vaccine Adjuvant

Vaccination is one of the most effective methods to prevent infectious diseases and manage healthcare costs 296. Traditional vaccines have good immunogenicity due to the complex nature of the formulation and the presence of adjuvants. However, purified preparations lack immunogenicity, which makes the addition of adjuvants essential. Adjuvants can simultaneously reduce the amount of antigen required, shorten the time needed for a protective threshold of antibody production and improve the intensity of the elicited responses, stimulate long-term memory responses to reduce the requirement of repeated vaccinations. Yingying Xu et al. 297 firstly reported the remarkable immunological adjuvant effect of AgNPs both *in vitro* and *in vivo* using model antigens ovalbumin and bovine serum albumin in 2013. After intraperitoneal or subcutaneous immunization of mice, AgNPs increased the production of serum antigen-specific IgG, as well as antigen-specific IgE, indicating that AgNPs stimulated Th2-biased immune responses. Further study of the mechanism of adjuvant revealed that AgNPs could recruit and activate local leukocytes and macrophages. Vahid Asgary et al. 298 evaluated AgNPs as an adjuvant for the rabies vaccine in 2014 and 2016, respectively. They found that although the load of AgNPs could significantly increase the immune responses by arising neutralizing antibody against rabies virus in mice, the lowest concentration of virus-loaded AgNPs decreased cell viability. This limited the use of AgNPs as an adjuvant in rabies virus. They then challenged the green synthesis of AgNPs using leaf extract of *Eucalyptus procera* and added AgNPs as an adjuvant in rabies veterinary vaccine, following by estimating vaccine efficacy in mice and dogs. They confirmed that the vaccine loaded with a suitable concentration of AgNPs was nontoxic 299.

#### Antidiabetic Agent

Diabetes mellitus (DM) is a group of metabolic diseases characterized by hyperglycemia. DM is due to either insufficient insulin secretion or insulin resistance of the cell. Commonly used hypoglycemic agents can lower blood sugar by promoting secretion of insulin or increasing cell sensitivity 300. In recent studies, it is noticed that AgNPs synthesized by plant extracts exhibit antidiabetic potential. Arumugam et al. 301 synthesized AgNPs using leaf extract of *Solanum nigrum* and evaluated the anti-hyperglycemic effect in alloxan-induced diabetic rats. They found that the blood glucose level of diabetic rats decreased when treated with AgNPs for 14 days and 21 days without significant acute toxicity. And they assessed glucose tolerance of AgNPs in diabetic rats. The results showed that AgNPs exhibited a good hypoglycemic effect compared to glibenclamide, a standard antidiabetic drug. Saratale et al. 4 demonstrated that AgNPs synthesized by leaf extract of *Argyreia nervosa* exhibited antidiabetic activity *via* inhibiting α-amylase and α-glucosidase. These two carbohydrate digestive enzymes contribute to decompose carbohydrates into monosaccharides. The antidiabetic mechanism of AgNPs is still unclear. Jihan Hussein et al. 302 hypothesized that AgNPs might influence insulin signaling pathway or insulin sensitivity in diabetic rats. The results supposed that AgNPs could activate protein kinase C and PI_3_K pathway at the insulin receptor substrate level, as well as inhibit protein kinase C isozymes, thus effectively enhance insulin secretion and sensitivity. It was highlighted that AgNPs were effective in reducing insulin resistance and DNA damage.

#### Biosensing and Imaging

Surface-enhanced Raman scattering (SERS) has attracted the attention of noble metals with Raman signals in many application strategies, including biochemical sensing, analytical chemistry, and materials science 303. Among these nanomaterials, AgNPs can be used as a cost-effective surface-enhanced Raman scattering substrate. Nanoparticles containing AgNPs can be used as biosensors to detect blood glucose, enzymes, molecular markers of tumor cells, pathogens, etc. For example, Jiang et al. 304 prepared silver-containing nanocomposites as acetylcholinesterase biosensors for electrochemical detection of organophosphorus pesticides. AgNPs improved the electrical conductivity and biocompatibility of nanocomposites and made them more suitable for enzyme activity and stability. Anderson et al. 305 prepared a high-sensitivity nonenzymatic biosensor for the detection of glucose using AgNPs as a conductive additive. Both the porous nanostructures of AgNPs and large surface areas of carriers enhanced the interaction sites between AgNPs and electrode/glucose, which could accelerate the electron transfer of AgNPs and therefore improve the sensitivity of the biosensor. Although the electrochemical characteristics and Raman scattering make AgNPs exhibit good application prospects in the field of biosensing, the matrix composition may affect their SERS and reduce the detection sensitivity. Therefore, it is necessary to modify AgNPs in order to improve the sensitivity of re-creating platforms. For example, Zeng et al. 306 synthesized hybrid Ag@NGO nanoparticles by a one-step reduction method. Among these platforms, the nanosized graphene oxide (NGO) worked as inert protective layers and provided an ultrathin protective layer for AgNPs. Ag@NGO exhibited the advantages of both SERS biosensing and drug delivery, ie, monitoring biomolecule signals in tumor cells through SERS biosensing and interacting with the anticancer drug doxorubicin through formation of π-π bonds. These results prove that AgNPs hold great application potential with capabilities of SERS biosensing.

Silver nanoclusters have unique optical and electrical properties and can be used as materials for synthetic probes. While proteins have multiple chelating and functional groups, therefore, they have unique advantages as ligands in biological imaging. Cunlan Guo and Joseph Irudayaraj 307 used denatured bovine serum albumin as a stabilizer to synthesize silver clusters, which could sensitively and selectively detect the content of mercury. The probe had important application value for detecting mercury content in water, soil and food. Sun et al. 308 used glutathione as a ligand to passivate silver nanoclusters and obtained highly sensitive fluorescent probes. During the passivation of glutathione, the specific recognition of silver nanoclusters modulated from Hg^2+^ to Cu^2+^. This fluorescent probe was highly sensitive and selective in detecting Cu^2+^ in blood samples. The synthesis of silver nanoclusters with DNA as the backbone has excellent spectral and photophysical properties. The generation of this fluorophore is highly dependent on the DNA sequence. Oligonucleotide sequence changes may trigger the adjustment of the photoluminescence emission band, thus identifying the mutant nucleotide sequence. Guo et al. 309 designed a double-stranded DNA scaffold that hybridizes probe DNA strands and sickle cell anemia mutation target DNA to generate fluorescent silver nanoclusters. The fluorescent silver nanoclusters specifically recognized sickle cell anemia mutations. The research extended from DNA scaffold single-stranded oligonucleotide to hybrid DNA double-stranded mutation site recognition, which may have more applications in the field of biological analysis. These studies suggest that silver nanoclusters have great clinical application potential.

## Potential Toxicity of AgNPs

**Figure 5:**
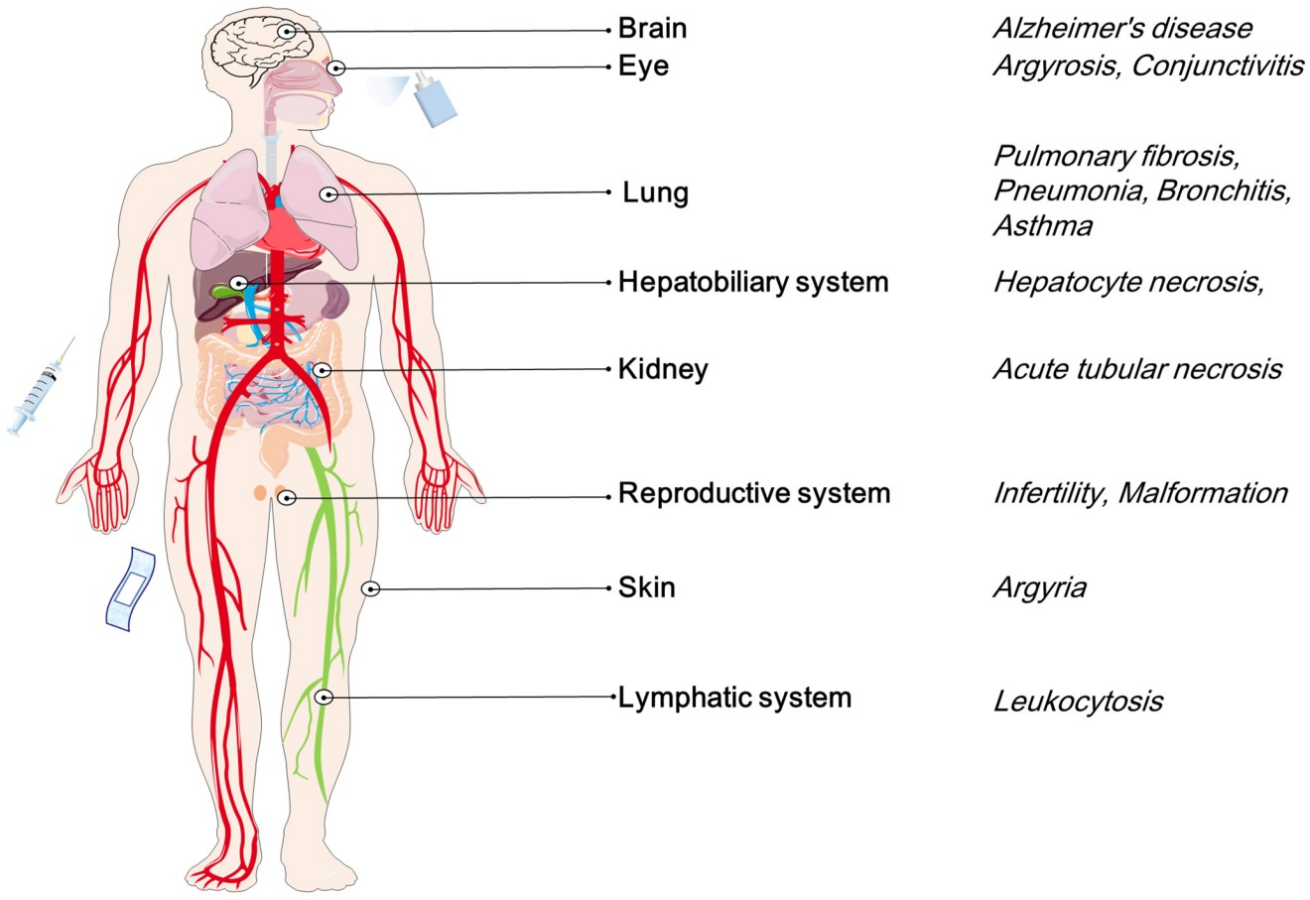
A schematic of potential toxicities of AgNPs in the human body. The exposure patterns of AgNPs include respiratory inhalation, intravenous injection and skin contact. Affected organs include the eye, kidney, skin, and nerves, respiratory, immune, hepatobiliary and reproductive systems. Diseases or pathologic changes induced by AgNPs are listed.

The potential harm of nanomaterials to organs and systems in the body has been gradually observed 310-312, which may influence the biomedical application of nanomaterial. Therefore, it is necessary to review the dynamics of AgNPs *in vivo*. AgNPs can be taken and distributed to different organs through a variety of routes of administration, mainly include inhalation, ingestion, skin contact, and subcutaneous or intravenous injection (**Figure 5**). The absorbed AgNPs are distributed in many systems 310, 311, such as the dermis, respiratory, spleen, digestive, urinary, nervous, immune and reproductive system, and mainly distributed in the spleen, liver, kidney and lung, while little deposition of AgNPs is observed in teeth and bones. The small-sized AgNPs are easy to penetrate the body and cross biological barriers like the blood-brain barrier and the blood-testis barrier, and subsequently induce potential cytotoxicity. Besides the directly exposed tissues, AgNPs can also be transported to different organs via blood circulation. Therefore, the non-specific distribution of AgNPs may produce cytotoxicities such as dermal toxicity, ocular toxicity, respiratory toxicity, hepatobiliary toxicity, neurotoxicity and reproductive toxicity, which limit the applications of AgNPs. The potential cytotoxicity of AgNPs depends on the routes of administration and the properties or characteristics of the AgNPs, such as the size, shape, and concentration. At the cellular level, Wang et al. 313 used TEM and integrating synchrotron radiation-beam transmission X-ray microscopy (SR-TXM) with 3D tomographic imaging to capture the information of the cellular uptake, accumulation, degradation, chemical transformation, and removal of AgNPs. The experiment revealed that the cytotoxicity was caused by the chemical transformation of AgNPs, ie. Ag^0^ transformed into Ag^+^, Ag-O-, and Ag-S- species, which might induce the cellular biochemical changes. However, there is still inadequate acknowledge of the potential cytotoxicity, long-term adverse health effects, and the specific mechanisms of AgNPs accumulated in the different tissues and organs. In order to develop AgNPs with better biocompatibility for medical applications, it is urgent to systematically study their potential cytotoxicity. This chapter provides a brief overview of the potential toxicity and possible mechanisms of AgNPs in different organs, including skin, eye, kidney, respiratory system, hepatobiliary system, central nervous system, immune system and reproductive system (**Table 6**).

### Skin Toxicity

Even as early as in 1614, Angelo Sala reported the first case of a definitive diagnosis of argyria, a kind of disease induced by the deposition of silver in tissues 314. Since the mid-19th century, it has been recognized that silver or silver compounds may induce some tissues to turn gray or blue-grey, especially involving the skin. The skin, as the largest organ and the first-line barrier of the human body, can isolate the external pathogens from the internal environment. Topically applied AgNPs may induce cytotoxicity in the site and penetrate the skin and subsequently access the systemic circulation followed by adverse effects on other organs. For example, applying AgNPs gel and covering dressings will allow particles to penetrate and accumulate in the skin and produce potential cytotoxicity 315. Before AgNPs, there are several reports on the skin toxicity of elemental silver, known as Argyria 316. Argyria is a disease characterized by permanent gray-blue pigmentation of mucous membranes, eyes and skin, occurring in individuals exposed to high concentrations of silver for a long period. G D DiVincenzo et al. 317 previously reported that the skin of workers exposed to silver aerosols showed a distinctive gray bluish hue change, and deposited silver was also detected in worker's hair, urine and feces. Jennifer et al. 318 reported a Argyria case. The patient showed uniform accumulation of silver on the skin after long-term consumption of silver solution. Current studies show that AgNPs can enter the hair follicles to induce local deposition and deposit into the subcutaneous structure by penetration pathways. The follicular penetration pathway is most commonly used to explain the penetration of particles into the skin 319-322. Yu Kyung Tak et al. observed that AgNPs of different shapes would remain at different layers of skin. Rod-shaped, spherical, and triangular AgNPs penetrated the dermis, epidermis and stratum corneum layers, respectively. They observed the behavior of AgNPs in subcutaneous capillaries. And prolonged exposure time would increase the amount of nanoparticles. Notably, they found that the penetration of AgNPs was achieved by the follicular penetration pathway and intercellular penetration pathway 323. Francesca et al. 324 attempted to use AgNPs to act on intact or damaged skin. They demonstrated a significantly higher penetration of AgNPs used on damaged skin as compared with intact skin, and they speculated that a small part of the particles would diffuse into the skin as silver ions. Radoslaw et al. 325 explored the cytotoxicity of AgNPs on epidermal keratinocytes (NHEK). The results showed that AgNPs inhibited cell proliferation and migration, induced activation of caspase 3 and caspase 7, and damaged DNA. In addition, by measuring the ATP content of cells treated with different concentrations (6.25, 12.5, 25 and 50 μg/ml), it was found that a high concentration of AgNPs significantly decreased the ATP production, and this phenomenon worsened with prolonging exposure time.

### Eye Toxicity

AgNPs agent may cause concentration-dependent acute conjunctival irritation, but there is still no reliable evidence for toxicological effects. Pattwat et al. 326 dripped 50 ppm and 2,5000 ppm colloidal AgNPs into the eyes of guinea pig and explored whether there were acute eye irritation or corrosion throughout the 78 hours observation period. Although transient mild conjunctival irritation, i.e. blood vessel hyperemia in conjunctivae, was observed within 24 hours after 5000 ppm AgNPs treatment, neither low-dose nor high-dose colloidal AgNPs caused any acute toxicological effects in guinea pigs. AgNPs may have developmental toxicity in the eyes of early-stage individuals, which can eventually result in multiple types of eye defects. Yuan Wu et al. 327 studied the developmental toxicity of AgNPs by using Japanese medaka at early-life stages as experimental models, including embryonic, larval and juvenile stages. The Japanese medaka was exposed to 100-1000 mg/mL AgNPs for 70 days and various morphological malformations were described and analyzed, such as edema, visceral deformities, heart malformations, spinal abnormality, especially eye defects. AgNPs-treated group showed different eye defects, such as microphthalmia, exophthalmia, cyclopia and anophthalmia. Histopathological examinations of 2-day-old larvae showed increased thickness of retinal pigment epithelium and missing of the retina in inner segments. Interestingly, comparing with the high-dose groups, the types and numbers of eye malformations in the low-dose groups were significantly higher. These morphological abnormalities and non-linear dose-response pattern suggest that the developmental toxicity of AgNPs may exhibit complex toxicological mechanisms.

### Respiratory toxicity

AgNPs can induce acute lung toxicity and therefore impair lung function, and the damage severity is related to particle accumulation and clearance. Akinori 328 et al. studied the pulmonary toxicity of nanometer particles in mouse models. Ultrafine particles may pass the air-blood barrier through the gap between alveolar epithelial cells, induce vacuolation and necrosis of bronchiolar epithelial cells, resulting in transient acute lung inflammation and tissue damage. The oxidative stress and apoptosis induced by ultrafine particles may contribute to lung damage. In addition, nanoparticles showed size-dependent pulmonary toxicity, i.e. the particles in smaller size exhibit higher capacity for inducing lung inflammation and tissue damage than larger size 36, 329. On the other hand, AgNPs may induce dose-dependent lung toxicity. Kaewamatawong et al. 330 demonstrated dose-dependent acute lung toxicity in mice induced by AgNPs using a single intratracheal instillation of 0, 10, 100, 1000 or 10000 ppm of colloidal AgNPs. And they observed moderate to severe bronchitis and multifocal alveolitis in 100, 1000 and 10,000 ppm AgNPs treated groups. Proinflammatory cytokines such as IL-1β and TNF-α released by alveolar macrophages and airway epithelial cells might involve in the inflammatory lesions in mice. The aggregation of AgNPs had a direct effect on the basement membrane, and disrupted equilibrium between the synthesis and degradation of the extracellular matrix, thus may cause pulmonary fibrosis. Similarly, they also speculated that AgNPs induced oxidative stress in the lung. Furthermore, they recognized that metallothionein (MT) expression induced by AgNPs might be regarded as one of the possible protective mechanisms of lung. Different concentrations of AgNPs, which induce lung damage, may also accumulate in peripheral organs and cause potential health risks. Joanna et al. 46 found that AgNPs disrupted the blood/alveolar epithelial permeability barrier, elicited oxidative stress, activated eosinophils and promoted the release of multiple cytokines. Most importantly, their results showed that AgNPs induced eosinophilic and neutrophilic inflammation, which was an important pathological change in asthma. This might suggest that exposure to AgNPs could trigger asthma.

### Hepatobiliary System Toxicity

Part of the ingested nanoparticles tend to be sequestered, degraded and accumulated in the liver, which means the liver may be responsible for the metabolism of nanoparticles as well as one of the most frequently attacked organs. On the other hand, the gallbladder collects, stores and excretes bile or biological waste to the intestine. Various metal nanoparticles, including AgNPs, are recognized to be exported from the liver through this pathway. Therefore, hepatocytes are widely studied in the liver toxicity of AgNPs. Maglie et al. 48 found that AgNPs induced severe hepatobiliary damage in mice, including significant hepatocyte necrosis and gallbladder hemorrhage. In this study, AgNPs exhibited size- and dose-dependent hepatobiliary toxicity, i.e. AgNPs in smaller size produced more serious toxic effects, and higher dose of AgNPs induced severer hepatobiliary damages. Camilla et al. 331 observed multi-system acute toxicities in mice with a single intravenous injection of AgNPs. First of all, significant hepatobiliary damages were recognized, including hepatocyte necrosis, micro-hemorrhage around the biliary tract, and portal vein injury. Secondly, they also observed that AgNPs could induce acute tubular necrosis and apoptosis, and moderate splenomegaly. The results of Mohammad et al. 332 showed that AgNPs penetrating via the skin induced time-dependent liver damage such as hyperemia, dilatation in central venous, swelling hepatocytes and increased inflammatory cells. Besides hepatocytes, Kupffer cells (KCs) are also responsible for the removal of AgNPs 333. KCs are macrophages that reside in the hepatic sinusoids and have the active ability of phagocytosis, maintaining the normal immune response and removing nanoparticles from organisms 333, 334. Therefore, KCs become the focus of research on liver toxicity and metabolism of AgNPs.

### Central Nervous System Toxicity

The central nervous system consists of two parts: the brain and the spinal cord. Lots of supporting non-nervous cells, i.e. neuroglial cells fill the interneuronal space within the central nervous system. In recent years, some articles point out that AgNPs may penetrate the brain and subsequently induce neuronal death. Due to the limited self-repairing ability of nerve cells, the potential neurotoxicity of AgNPs is receiving more attention. Different exposure patterns can lead to the accumulation of AgNPs in the brain. Injected AgNPs cross blood-brain barrier (BBB) and then penetrate the brain, while inhaled AgNPs reach the central nervous system through the olfactory and/or BBB 335, 336. Due to the unique physicochemical properties of AgNPs, deposited AgNPs in nerve cells, astrocytes and extravascular lymphocytes may cause and aggravate neurotoxicity and inflammation, and increase the permeability of BBB. In the study of the cytotoxicity of AgNPs on rat cerebral astrocytes, Cheng et al. 337 investigated the neurotoxicological effects of AgNPs and Ag^+^ and compared the mechanisms. Both AgNPs and Ag^+^ exposure could internalize silver in astrocytes in dose- and time-dependent manners. The AgNPs had higher bioaccumulation ability than Ag^+^ after 24 h treatment. After the same treatment time, they found that AgNPs might induce intracellular ROS generation in rat cerebral astrocytes and caused cell apoptosis, however, there were undetectable alterations in Ag^+^ group. More importantly, they confirmed that AgNPs could increase the level of phosphorylated JNK, a kind of kinase involved in mediating apoptosis. The non-cytotoxic dose of AgNPs, rather than Ag+, might induce neuroinflammation by promoting the secretion of multiple cytokines of astrocytes, including CINC-2a/b, CINC-3, IL-10, IP-10, L-selectin and thymus chemokine. Liming et al. 38 investigated the neurotoxicity of AgNPs in the rat after intragastric administration of low-dose (1 mg/kg, body weight) or high-dose (10 mg/kg, body weight) for two weeks. They observed a variety of cell morphological changes in the nervous system, including neuron shrinkage, astrocytes swelling and extravascular lymphocytes. They also observed significantly increased inflammatory factors such as IL-4 in the serum. These data supported the proinflammatory effects of AgNPs in the nervous system. Then they focused on the possible mechanisms for AgNPs or Ag^+^ transporting across the blood-brain barrier. AgNPs or released Ag^+^ might cross through the blood-brain barrier via ionic pores or channels and subsequently damage the nerve cells. Besides, AgNPs could enter the central nervous system via vesicular transport of endothelial cells and subsequently induced neuroinflammation. At the same time, they demonstrated the increased permeability of the blood-brain barrier in a rat model after AgNPs treatment. They also observed that AgNPs might inhibit the antioxidant defense of astrocytes by increasing thioredoxin interacting protein, thus lead to the central neurotoxicity. AgNPs might induce ROS, inflammation and apoptosis through regulating the MAPK pathway, mTOR activity and Bcl-2 expression in astrocytes. AgNPs could cause severe ultrastructural changes in astrocytes, including mitochondrial contraction, endoplasmic reticulum expansion and nuclear atypia. Furthermore, AgNPs regulated the expression of multiple genes, inhibited metabolic and biosynthetic processes, thus affect astrocytes function and increase the neurotoxicity. More importantly, the impairing of learning, memory and cognition processes by AgNPs reduced the learning ability and cognition function of rats 338. AgNPs may induce neurological diseases such as Alzheimer's disease by altering gene expression. Chin et al. 339 reported that AgNPs could induce the expression of amyloid precursor protein (APP) gene in nerve cells. APP gene promoted the deposition of amyloid-β (Aβ) protein, a key pathological feature of Alzheimer's disease.

### Kidney Toxicity

The kidney participates in balancing body fluid volume and pH, regulating osmotic pressure and electrolyte concentration, drug metabolism, and toxic emissions. Abnormal renal function may occur in AgNPs-treated mammalian kidneys. AgNPs exhibits a dose-dependent accumulation in most examined tissues, such as the brain, lung, liver, dermis, blood and testes. However, there is a gender-related difference in silver accumulation in the kidney. Wan et al. 340 observed that female rats treated with AgNPs had a twofold higher concentration of silver in kidneys than male rats. Ag enhancement staining of the kidneys showed that AgNPs preferentially accumulated in the basement membrane of the glomerulus as well as renal tubules, while mildly accumulated in the adrenal capsule and cortex. There were two possible mechanisms of gender difference in the accumulation of AgNPs: the gender difference in the expression of organic cation transporters, and hormonal regulation. Renal metallothionein and zinc-binding protein, a kind of transporter or binding protein molecules in the kidney, might contribute to the silver accumulation. While organic anions secreted by kidneys might influence the clearance and accumulation of silver 341. Mirta et al. 342 studied the uptake mechanism and potential cytotoxicity of AgNPs in porcine kidney (Pk15) cells* in vitro*. TEM results showed that there were aggregates in the lysosome and early endosomes. In addition to micropinocytosis, as an uptake pattern, clathrin- and caveolin-mediated endocytosis could also be the possible endocytotic mechanisms. AgNPs could decrease the number of viable Pk15 cells *in vitro* in a dose-dependent manner. Hua et al. 343 studied the distribution, accumulation and potential toxicity of AgNPs in different sizes in liver, lung and kidney of mice. They found that AgNPs could be taken up by vascular endothelial cells, then induced the generation of intracellular ROS and down-regulated the expression of vein endothelial cadherin. Therefore, AgNPs destroyed the conjunction between endothelial cells, allowing AgNPs to cross the endothelial layer and accumulate in organs. Besides, the leaking AgNPs could also result in peripheral inflammation in a size-dependent manner. Mice receiving single or multiple intravenous injections of AgNPs showed basement membrane injury in glomeruli.

### Immune System Toxicity

Our immune system, a natural host defense barrier, is composed of immune cells, tissues and organs, can constantly interact with the internal environment and protect us from pathogens in the external environment, and provide the inherent knowledge to separate the friend and foe within our body 344. Seung et al. 345 found that AgNPs inhibited the proliferation and the production of cytokines, including IL-5, INF-γ and TNF-α, and induced cytotoxicity in peripheral blood mononuclear cells in a concentration-dependent manner. AgNPs may deposit in the immune organs and influence the number of immune cells and the production of cytokines. Wim et al. 45 investigated the effects of AgNPs on the immune system of rats by repeated intravenous administration of AgNPs with different sizes (20 nm and 100 nm) for 28 days. They found that AgNPs administered at the maximum dose (6 mg/kg) were still well tolerated by the rats. The size and weight of the spleen increased significantly, possibly due to the increased cell number of T cells and B cells. However, the cytotoxic activity of NK cells in the spleen was almost completely inhibited. For multiple immune-related cytokines in serum, levels of interferon-γ, IL-10, IL-6 and TNF-α declined, while levels of IL-1β, IgM and IgE increased. The number of neutrophilic granulocytes in peripheral blood also increased. Besides, brown and black pigments were observed in histopathological sections of spleen and lymph nodes, indicating the accumulation of AgNPs in these immune organs. This study suggested that the immune system was sensitive to the potential adverse effects of AgNPs. The spleen may be one of the main organs for the accumulation and elimination of AgNPs, and both processes are in a sex-dependent manner. Yuying et al. 346 observed the potential acute toxicity and biokinetics after repeated intravenous administration of AgNPs in mice. During the 14-day observation period, both the survival and behavior of the mice were normal. They found that AgNPs were widely distributed in tissues, especially in the spleen, followed by the liver. The biokinetics of AgNPs in the kidney and lung seem to show gender-related differences, i.e. the accumulation of silver in kidney and lung of female mice was higher than that of male mice, the longer elimination half-life and slower clearance of AgNPs in female mice than male mice. Besides, the KCs in the liver were mainly responsible for the retention and elimination of AgNPs. The silver content in the liver significantly decreased after one day. While in the spleen, the marginal zone and the red pulp macrophages contributed to the clearance of silver.

### Reproductive System Toxicity

Biological barriers, such as the blood-testis barrier, placental barrier and epithelial barrier, can protect the reproductive system from infection and toxicity. AgNPs can cross the biological barriers to deposit in reproductive organs including testis, epididymis, ovary and uterus. Thus, AgNPs may damage germ cells and related cells, such as primary and secondary follicles, germline stem cells, Sertoli cells and Leydig cells 42, 347. Besides, AgNPs can also cause changes in sexual behavior by affecting the secretion of hormones within the reproductive organs and glands. Further studies confirmed that the reproductive toxicities of AgNPs are achieved by increasing inflammation, disrupting DNA structure, down-regulating gene expression, decreasing mitochondrial function, inducing ROS production and apoptosis. These toxicities of AgNPs to the reproductive system are size-, time- and dose-dependent 347, 348. Zhang et al. 347 investigated the effects of AgNPs with different sizes (10 nm and 20 nm) on male somatic Leydig cells and Sertoli cells, and found that cell viability was inhibited by AgNPs in size- and concentration-dependent manners. The 10 nm AgNPs showed more cytotoxicity than the 20 nm AgNPs. and cell proliferation was significantly decreased as the concentration of AgNPs increased from 0 to 100 μg/ml. AgNPs-treated Sertoli cells showed decreased mRNA levels of ZO-1 and Cx43, both are involved in encoding tight junction proteins which playing a crucial role in the formation of BTB. As well as AgNPs-treated somatic Leydig cells showed decreased mRNA levels of StAR, 3β-HSD and 17β-HSD, which are involved in the production of testosterone. It is widely acknowledged that spermatogonial stem cells (SSCs) can continuously proliferate, renew and produce sperms throughout male's postnatal life. Cytokines secreted by Sertoli cells play an important role in the proliferation and renewal of SSCs. In this study, AgNPs-treated Leydig cells secreted decreased level of testosterone, which was responsible for inducing spermatogenesis and maintaining normal functions of Sertoli cells. These results suggest that AgNPs can impair the function of Leydig cells and Sertoli cells, then worsen the function of SSCs, ultimately suppress male fertility. Cynthia et al. 42 evaluated the fecundity and development of Drosophila fed with AgNPs at various concentrations from 0 to 5 µg/mL. AgNPs decreased the viability and delayed the development of Drosophila in a dose-dependent manner. Germline stem cells (GSCs) and early germ cells were concentrated at the apical tip of the testis. Among different treated groups, a significantly increased ROS level was observed at this tip area of Drosophila treated with 5 µg/mL AgNPs. They also proved that AgNPs might disrupt GSCs maintenance by triggering precocious differentiation of GSCs, thereby decreased the number of sperms. Besides, the first generation of Drosophila fed with a higher concentration of AgNPs showed delayed eclosion and decreased male offsprings as compared to control or lower concentration group. The mating success of Drosophila and the number of their second or third generations decreased in AgNPs-treated groups than the control group. This might suggest that AgNPs accumulated in GSCs could be passed onto offspring and affect the development and fecundity of the offspring. Lafuente et al. 37 studied parameters of epididymal sperm of rat fed with different doses of PVP-AgNPs (50, 100 and 200 mg/kg/day), including sperm morphology, motility and viability. PVP-AgNPs induced sperm morphology abnormalities in a dose-dependent manner. In their study, 100 mg/kg/day of PVP-AgNPs significantly increased abnormal morphologies in epididymal sperms, such as banana head, tail bending, head loss and neck abnormalities. Abnormal sperm morphology reduced sperm motility and vitality. Some studies focus on the effects of AgNPs on female reproduction. Chen et al. 349 evaluated the potential toxicity of AgNPs and Ag^+^ on zebrafish oocytes. Vacuolation or swollen mitochondria, and condensed nucleus were observed in AgNPs- and Ag^+^-treated follicular cells. Zebrafish oocytes treated with AgNPs or Ag^+^ showed a decreased concentration of cAMP, which plays a key role in the maintenance of meiosis arrest, and results in meiosis resumption and subsequent oocyte maturation. Besides, AgNPs and Ag^+^ up-regulated caspase 3 and caspase 9, respectively, both of which play important roles in the initiation and execution of apoptosis, ultimately leading to apoptosis in ovarian follicle cells.

## Conclusion and prospect

Over decades, AgNPs have been studied rapidly and extensively due to the unique physical, chemical, optical, electronic and catalytic properties. These properties are closely related to characteristics of AgNPs, especially the size and shape. AgNPs with different characteristics can be produced by physical, chemical and biological routes. External energy sources such as light, heat, electricity, sound and microwave can be used in the synthesis process. Various factors should be considered in the synthesis of AgNPs with expected size and shape. Besides the types of precursor salts, additives such as reducing agents, capping agents and stabilizers, as well as the importance of reaction parameters, including reaction temperature, time, pH and extra energy sources should be recognized in the production process. Among these methods, biological synthesis using bacteria, fungi and plant extract proves a simple, environmentally friendly, cost-effective and reliable approach. Compared with physical and chemical methods, biological method does not require high temperature or toxic/hazardous additives, but the potential pathogens need to be carefully considered. We review the synthesis methods of AgNPs and compare the advantages and disadvantages to help understand how to obtain nanoparticles with controlled size and shape.

AgNPs have broad prospects in medical applications. Among them, antimicrobial and anticancer properties have received more attention. A variety of factors influence the antimicrobial and anticancer effects of AgNPs, including size, concentration/dose, exposure time, stabilizer and surface charges. The proposed mechanisms for antimicrobial activity of AgNPs involve destroying the structure of cell wall, inducing ROS production and DNA damage. Anticancer mechanisms of AgNPs are more complicated. AgNPs can induce apoptosis and necrosis of cancer cells by destroying cell ultrastructure, inducing ROS production and DNA damage, inactivating proteins and regulating multiple signaling pathways. Besides, AgNPs may block invasion and migration of cancer cells by inhibiting angiogenesis within the lesion. However, the potential cytotoxicity of AgNPs may limit their medical applications. In order to improve the compatibility of AgNPs, proper surface functionalization is widely concerned. The AgNPs surface allows coordination of multiple ligands and thus can be functionalized. The surface functionalization of AgNPs can simultaneously improve their biological safety and challenge their drug delivery, which is conducive to the development of more antibacterial and antitumor agents involving AgNPs. AgNPs can also be used as an additive or adjuvant in bone scaffolds, dental materials and vaccines. The antidiabetic effect of AgNPs is also explored. Besides the impressive antimicrobial and anticancer activities, the unique optical properties of AgNPs make them great clinical potential in the field of biosensing and imaging. The AgNPs surface allows coordination of multiple ligands and thus can be functionalized.

Although most studies focus on the therapeutic purposes of AgNPs, the potential toxicities of AgNPs in multiple systems including skin, eyes, kidney, respiratory system, hepatobiliary system, immune and reproductive systems have been discussed. Further in-depth studies are required to evaluate the biocompatibility and potential cytotoxicity of AgNPs, which may help to develop safer and biocompatible AgNPs-based agents.

In this review, we separately introduce the synthesis method and anticancer properties of Ång-scale silver particles in the relevant sections. Compared with AgNPs mentioned in this review, we prepared pure and fine silver particles with Ångstrom size. This ultra-fine size may be a threshold for silver particles in the medical applications, that is, Ång-scale silver particles exhibit broad-spectrum anticancer activities without obvious cytotoxicity. This exciting discovery inspires us to explore more promising applications of Ång-scale silver particles in nanomedicine.

## Figures and Tables

**Table 1:** Synthesis of Silver Nanoparticles by Physical Methods

Method	Silver precursor	Stabilizer/Surfactant/Dispersant	Operating conditions	Size (nm)	Shape	Reference
Ball milling method	Silver powder	-	Dry, under protective Ar gas atmosphere, below -160 ± 10°C	4-8	Spherical	85
	Silver wire	-	Multi-walled carbon nanotubes-aqueous nanofluids, 15-40°C, DC power	About 100	Spherical	86
Electrical arc-discharge method	Silver wire	-	25°C, current, voltage, deionized water	-	-	66
	Silver wire	-	DC arc-discharge system, 70°C, stirring	72	Spherical	63
	Silver wire	-	DC arc-discharge system, room temp.	19	Cubic	87
	Silver wire	-	DC arc-discharge system, deionized water, stirring	20-30	Spherical	61
Laser ablation method	Silver plate	-	Laser pulses, organic solvent	4-10	Spherical	88
	Silver plate	PVP	Laser pulse, stirring	20-50	Spherical	65
	Silver plate	-	Laser pulse, solution of chlorobenzene, stirring	25-40	Spherical	89
Physical vapour condensation	Silver wire	Fructose	High voltage power, rapid cooling	19.2±3.8 Ång	Spherical	21

**Table 2:** Synthesis of Silver Nanoparticles by Chemical Methods

Method	Silver precursor	Reducing agent	Stabilizer/Surfactant/Dispersant	Operating conditions	Size (nm)	Shape	Reference
Chemical reduction	Tollens reagent	Triazole sugar	-	Room temp.	9.7 ± 1.9	Spherical	72
	AgNO_3_	Sodium citrate and tannic acid	-	Room temp., 100°C	About 30	Spherical	90
	AgNO_3_	Trisodium citrate/sodium borohydride/ascorbic acid	Sodium borohydride	Heat	-	-	91
	AgNO_3_	Hydrazine hydrate	Sodium dodecyl sulphate	Room temp.	40-60	Spherical	92
Photochemical method	AgNO_3_	Sodium borohydride	Trisodium citrate	Room temp., LED of specific wavelength	40-220	Decahedron, plate, prism	93
	AgNO_3_	NaCl	-	Room temp., UV light, stirring	About 8.6	Spherical	13
	AgNO_3_	Sodium borohydride	Trisodium citrate	Mixed light irradiation, DC power	31.4 ± 1.4	Triangular plate	53
	AgNO_3_	2-hydroxy-2-methylpropiophenone	-	Polychromatic Xe-Hg lamp, stirring	0.74-1.12	Spherical	79
	AgNO_3_	Sodium borohydride, tri-sodium citrate dihydrate	Polyvinylpyrrolidone	LED of different wavelength	4-20	Spherical, rod, polyhedrons	94
	AgNO_3_	Sodium citrate	-	25°C, Hg-halide floodlight	4.92±1.17	-	95
Electrochemical method	Silver plates	-	-	Room temp., galvanostatic	20	-	96
	Ag electrodes	-	-	20-95°C, 20 V, direct current, stirring	2-20	Spherical	80
	Ag electrodes	-	N-vinyl-2-pyrrolidone and sodium lauryl sulfate	Room temp., alternating polarity, 5-10 mA direct current, stirring	10-55	Spherical	97
	Silver plate	-	Chitosan	25°C, constant potential, UV irradiation, stirring	2 - 16	Spherical	98
	AgNO_3_	Sodium borohydride	Chitosan	Room temp., voltalab potentiostat/galvanostat	About 50	-	69
Microwave-assisted synthesis	AgNO_3_	Apple extract	-	Microwave, 100 ℃	22.05 ± 1.05	Elongated and spherical-like	99
Sonochemical method	AgNO_3_	Glucose	Gelatin	High-intensity ultrasound irradiation, ambient conditions	About 5.3	Spherical	84
	AgNO_3_	-	J-carrageenan	Room temp., ultrasound irradiation	7.07 ± 2.54; 4.08 ± 2.09; 5.01 ± 6.48	Spherical	12
	AgNO_3_	-	Dihydrolipoic acid	Ultrasound irradiation, 50°C	5-10	Nanocluster	100
	AgNO_3_		Dihydrolipoic acid	Ultrasound irradiation, UV, room temp.	2 - 4	Nanocluster	101
	AgNO_3_	Polyacrylic acid	Acrylate	Ultrasound irradiation, 50°C	10-15	Spherical	102
	AgNO_3_	Sodium borohydride	Poly(vinyl alcohol)	Ultrasound irradiation, 60°C	13, 15, 18	Spherical	103
	AgNO_3_		Dihydrolipoic acid	Ultrasound irradiation, room temp.	2-3; 5-10	Nanocluster	104

**Table 3:** Bacteria-, Fungi-, Algae-mediated Synthesis of Silver Nanoparticles

Bacteria/Fungi/Algae	Position	Precursor	Responsible organic components/functional groups	Operating condition	Size (nm)	Shape	Reference
*Streptomyces violaceus*	Extracellular	AgNO_3_	Exopolysaccharide	37°C; shaking; pH 7.0;	10-60	Cubic; crystalline; spherical	132
*Penicillium polonicum*	Extracellular	AgNO_3_	Proteins	Room temp.; shaking; light	10-15	Spherical; near spherical	133
*Falcaria vulgaris*	Extracellular	AgNO_3_	Hydroxyl group	50°C	10-30	Spherical	134
*Pseudomonas*	Extracellular	AgNO_3_	Aromatic and aliphatic amines	28°C; shaking	10-40	Irregular	135
*Pantoea ananatis*	Extracellular	AgNO_3_	Proteins or amino acids	37°C; shaking	8-90	Spherical	136
*Fusarium oxysporum*	Extracellular	AgNO_3_	Proteins	28°C; shaking	21.3-37.3	Spherical; oval	111
*Botryosphaeria rhodina*	Extracellular	AgNO_3_	NADH-dependent nitrate reductase	Room temp.; dark	below 20	Spherical	137
*Monascus*	Extracellular	AgNO_3_	Lactone ring	28-30°C; shaking	10-30; 15-40	Spherical	138
*Aspergillus tamarii*	Extracellular	AgNO_3_	NADH-dependent nitrate reductase	25±2°C; shaking	3.5 ± 3	Spherical	120
*Nostoc linckia*	Extracellular	AgNO_3_	Phycocyanin	Room temp.; pH 10.0	9.39-25.89	Spherical	139
*Caulerpa serrulata*	Extracellular	AgNO_3_	Caulerpenyne; caulerpin	27-95°C; pH 4.1-9.5	10 ± 2	Crystalline; spherical	125
*Laurencia aldingensis*	Extracellular	AgNO_3_	Proteins	Dark; shaking	5-10	Spherical	140

**Table 4:** Plant-mediated Synthesis of Silver Nanoparticles

Plant	Plant part	Precursor	Responsible phytoconstituent	Operating condition	Size (nm)	Shape	Reference
*Coptis chinensis*	Leaf	AgNO_3_	-	Room temp.; dark	6-45	Spherical	23
*Phyllanthus* *pinnatus*	Stem	AgNO_3_	Phytochemicals	Room temp.; sterility	below 100	Cubical	141
*Parkia speciosa*	Leaf	AgNO_3_	Polyphenols	60°C; pH 11.0	26-39	Spherical	142
*Plantago major*	All	AgNO_3_	-	85°C; dark	10-20	Spherical	130
Avicennia marina	Leaf, stem and root	AgNO_3_	-	Room temp.; shaking	About 75	Spherical	131
*Origanum vulgare L.*	Aerial part	AgNO_3_	-	-	2-25	Cubic	143
*Gossypium hirsutum*	Shoot	AgNO_3_	-	60°C; shaking	20-100	Spherical	144
*Flacourtia indica*	Leaf	AgNO_3_	Phenolic, lignin and sterols	50°C	14-24	Spheroid	145
*Walnut*	Fruit	AgNO_3_	-	37-40°C; shaking; dark	About 31.4	Spherical	128
*Cleome viscosa L.*	Fruit	AgNO_3_	-	Room temp.; dark	20-50	Spherical	146
*Alpinia katsumadai*	Seed	AgNO_3_	Phytochemicals	Room temp.; shaking; dark; pH 10	About 12.6	Quasispherical	147
*Ocimum Sanctum*	Leaf	AgNO_3_	Quercetin	-	250-600	Spherical	129
*Mimosa Pudica*	Root	AgNO_3_	-	Room temp.	35-42.5	Spherical	148
*Aloe vera*	Leaf	AgNO_3_	Lignin, hemicellulose, and pectins	100°C or 200°C; shaking	70.70 ± 22, 192.02 ± 53	Spherical	149
Carambola	Fruit	AgNO_3_	Polysaccharide and ascorbic acid	Stirring at 40°C	10-40	Face-centered-cubic	150
*Anogeissus latifolia, Boswellia serrata*	Gum ghatti, gum olibanum	AgNO_3_	-	121°C, 15 psi	5.7 ± 0.2; 7.5 ± 3.8	-	151

**Table 5:** Anticancer Mechanisms of Silver Nanoparticles

Cancer cell lines	AgNPs		Concentration, IC_50_, exposure time	Manners	Mode of action	References
	Synthesis methods	Size; Shape				
HeLa cells	Plant	40 nm; spherical and pentagonal	25, 50, 100, 250 μg/mL; 24 h	Dose-dependent	ROS generation; ultrastructural changes; mitochondrial dysfunction	229
HeLa cells	Plant	33 nm; face-centered-cubic	0-100 μg/ml; 24 h	Concentration-dependent	Sub G1 cell cycle arrest; ROS generation; down-regulation of MMP	261
HeLa cells	Chemical	20-40 nm; spherical	1.35 μg/mL and 13.5 μg/mL; 24 h and 48 h	Dose-, concentration- and time-dependent	Decreased the number of cells at S and G2/M phase; increased the number of cells at sub-G1 phase	244
HeLa cells	Chemical	26.5 ± 8.4 nm; spherical	10, 20, 50 μg/mL; 10 h and 24 h	Dose- and time-dependent	Regulation of PtdIns3K signaling pathway	22
A549	Fungi	25 nm; round and triangular	1-10 μg/mL; 48 h	-	ROS generation; nucleus damage	273
A549	Plant	17-25.8 nm	25 µg/ml	-	Activation of apoptotic gene; inhibition of cell migration and invasion	274
MCF-7	Plant	22 nm; spherical	IC_50_: 20 µg/ml; 24 h and 48 h	Dose- and time-dependent	ROS generation; DNA damage; disruption of the cell membrane	275
MCF-7	Plant	12 nm; different shapes	IC_50_: 20 μg/mL; 24 h	Dose-dependent	Regulation of Bax and Bcl-2 gene expression	276
MCF-7	Peptides	31.61 nm; spherical	IC_50_: 104.1 μg/mL; 24 h	Dose-dependent	ROS generation; disruption of mitochondrial respiratory chain	277
MCF-7, EAC	Algae	7.1-26.68 nm; spherical	IC50: 13.07 ± 1.1 µg/mL; 48h	Dose-dependent	Inhibition of proliferation; mitochondria dysfunction	278
A549	Plant	6-45 nm; spherical	10 µg/mL and 25 µg/mL; 24h	Dose-dependent	Inhibition of proliferation, migration and invasion	23
MCF-7; MDA-MB-231	Plant	15-30 nm; spherical	IC_50_: 20 μg/mL (MCF-7), 30 μg/mL (MDA-MB-231)	Dose-dependent	Regulation of p53, Bax and Bcl-2 expressions	279
A549	Plant	45.12 nm; spherical	IC_50_: 62.82 μg/mL (24 h) and 42.44 μg/mL (48 h)	Dose- and time-dependent	S phase cycle arrest; decrease of cell population in sub G1 phase	280
A549; Hep G2	Purchased	21 ± 8 and 72 ± 11 nm; spherical	1-20 μg/mL; 48 h	Concentration- and dose-dependent	Inhibition of telomerase activity and telomere dysfunction	240
HT29	Plant	9.13 ± 4.86 nm; spherical	IC_50_: 38.55 μg/mL; 24 h	Dose-dependent	Induction of apoptosis pathway	281
HCT116	Bacteria	15 nm; spherical	IC_50_: 0.069 µg/mL; 24 h	Dose- and time-dependent	Induction of nuclear condensation and fragmentation	159
HCT-116	Plant	24-150 nm; spherical, triangular	IC_50_: 100 μg/ml; 24 h	Dose-dependent	Up-regulated modulators of apoptosis, Caspase-3, Caspase-8 and Caspase-9; mitotic arrest; DNA fragmentation	282
PANC-1	Purchased	2.6 and 18 nm; spherical	IC_50_: 1.67 μg/mL (2.6 nm), and 26.81 μg/mL (18 nm); 1 h, 24 h	Size- and concentration-dependent	Ultrastructural change; regulation of p53, Bax, Bcl-2, RIP-1, RIP-3, MLKL and LC3-II expression,	211
SCC-25	Purchased	10 ± 4 nm; spherical, cubic	IC_50_: 37.87 μg/mL; 24 h	Dose-dependent	Chromosome instability; mitotic arrest; regulation of gene expression	24
HOS; HCC	Fungi	8 ± 2.7 nm; spherical	IC_50_: < 5 μg/mL (Huh7 cells), 10 μg/mL (OS cells)	Dose-dependent	ROS generation; activation of JNK signaling	283
CNE; HEp-2	Chemical	20 nm; spherical	IC_50_: 9.909 μg/mL; 24 h	Dose-dependent	Mitotic arrest; regulation of Bax and P21 and Bcl-2 expression	284
PC-3	Plant	9 - 32 nm; spherical	IC_50_: < 10 μg/mL; 24 h	Dose-dependent	Decrease of stat-3 and bcl-2 expression; increase of caspase-3 expression	285
DU145; PC-3; SKOV3; A549	Plant	10 - 30 nm; spherical	IC_50_: 4.35 μg/mL (DU145); 7.72 μg/mL (PC-3); 4.2 μg/mL (SKOV3); 24.7 μg/mL (A549)	Dose-dependent	ROS generation; regulation of LPO and GSH level; regelation of caspase, p53 and Bax and Bcl-2	29
DLA	Bacteria	50 nm; spherical	IC_50_: 500 nmol/L; 6 h	Dose- and time-dependent	Activation of caspase 3; DNA fragmentation	286
SKBR3; 8701-BC; HT-29; HCT 116; Caco-2	Bacteria	11 ± 5 nm; spherical	IC_50_: 5 μg/ml (SKBR3); 8 μg/ml (8701-BC); 20 μg/ml (HT-29); 26 μg/ml (HCT116); 34 μg/ml (Caco-2)	Dose- and time-dependent	Decrease of MMP-2 and MMP-9 activities; ROS generation	269
Murine fibrosarcoma	Chemical	10 nm; spherical	IC_50_: 6.15 mg/kg	Dose-dependent	ROS generation; alteration of the IL-1b function	287
BxPC-3; A549; PC-3; Hep G2; CNE1; AsPC-1; U-87 MG; SW480; EC109; MDA-MB-231	Physical	19.2 ± 3.8 Ång; spherical	IC50: 10.36-25.85 μg/ml; 0 - 400 min, 24h	Dose- and time-dependent	Ultrastructure change; ROS generation; mitochondrial dysfunction; cell cycle arrest	21

*NOTE: ROS, reactive oxygen species; MMP, matrix metalloproteinase; LPO, lipid peroxidation; GSH, glutathione; JNK, c-jun N-terminal kinase; MCF-7, human breast cancer cell line; EAC, ehrlich ascites carcinoma; A549, human lung carcinoma cells; BxPC-3, human pancreas adenocarcinoma cells; PC3, prostate adenocarcinoma cells; HepG2, hepatocellular carcinoma cells; CNE1, nasopharyngeal carcinoma cells; AsPC-1, pancreas adenocarcinoma cells; U-87 MG, glioblastoma cells; SW480, colorectal adenocarcinoma cells; EC109, esophageal cancer cells; MDA-MB-231, breast adenocarcinoma cells; HT29, human colorectal adenocarcinoma cell line; HCT-116, human colon cancer cell line; PANC-1, human pancreatic ductal cell line; SCC25, human tongue squamous carcinoma; DU145 and PC-3, human prostate carcinoma cell lines; SKOV3, human ovarian carcinoma; CNE, human nasopharyngeal carcinoma cell line; HEp-2, laryngeal carcinoma cell line; DLA, Dalton's lymphoma ascites cell lines; SKBR3, human breast cancer cell line; Caco-2, heterogeneous human epithelial colorectal adenocarcinoma cells; HCC, human hepatocellular carcinoma cells; HOS, human osteosarcoma cells; MDA-MB-231, triple-negative breast cancer cell line.

**Table 6:** Potential toxicity of AgNPs* in vivo* and *in vitro*

Objects			Exposure		Toxicity				References
Animal model	*In vitro/vivo*	Cell lines/Tissues	Size; Shape	Dosages	Route	Time	Effect	Toxicity manners	
Pig	*In vitro* and *vivo*	HEKs and porcine skin	20, 50 and 80 nm	0.34, 1.7 μg/mL	Incubation; skin contact	Acute: 18 and 24 h; chronic: 14 d	Focal inflammation	Dose-dependent	350
Mice	*In vivo*	Liver	Less than 30 nm	10 ppm	Skin contact	2, 7 and 14 d	Central venous dilation; hyperemia, cell swelling, Kupffer and inflammatory cells increase	Time-dependent	332
Mice	*In vivo*	Spleen, liver, lung and kidney	12-20 nm	7.5, 30 and 120 mg/kg	Intravenous administration	7 and 14 d	Induction of inflammatory reactions in lung and liver cells	Gender-, concentration- and time-dependent	346
Mice	*In vivo*	Lung	10-20 nm; spherical	10, 100, 1000 and 10,000 ppm	Intratracheally administration	1, 3, 7 and 15 d	Acute lung inflammation and bronchitis; hyperplasia of alveolar epithelial cells	Dose-dependent	330
Mice	*In vivo*	Liver, spleen, kidneys, heart, lungs, testes, stomach, intestine and seminal vesicles	3±1.57 nm; spherical	11.4-13.3 mg/kg	Intravenous injection	1, 28 d	Inflammatory response; alteration of hematological factors; change of gene expression; ROS generation	Dose-dependent	351
Mice	*In vivo*	Liver, kidneys and lung	10, 75 and 110 nm; spherical	25 μg/mice	Intravenous administration	1, 3 and 7 d	Peripheral inflammation in liver, kidneys and lungs	Time-, concentration- and size-dependent	343
Mice	*In vivo*	Lung	20 and 110 nm	0.05, 0.15, 0.45 and 1.35 mg/kg	Intratracheal instillation	1, 7 and 21 d	Alter SP-D level; deficit immune defense function	Size- and stabilization-dependent	44
Mice	*In vivo*	Brain, lung, liver, kidney and testis	22, 42 and 71 nm	0.25 mg/kg, 0.50 mg/kg, 1.00 mg/kg	Oral administration	14 and 28 d	Induce organ toxicity and inflammatory responses	Dose-dependent	352
Mice	*In vivo*	heart, lung, kidney, liver and blood	1.4-250nm	11.4-13.3mg/kg body weight	Intravenous administration	28 d	Induce gene expression; ROS generation; apoptosis	Dose-dependent	351
Mice	*In vitro* and* in vivo*	A549, BxPC-3; PC-3; Hep G2, CNE; AsPC-1; U-87 MG; SW480; EC109; MDA-MB-231; VSMC; HMEC; LO2; 293FT; tumor, brain, heart, kidney, lung, spleen, and liver	19.2±3.8 Ång, spherical or ellipsoidal	0-32ng/µl, 1.875 mg/kg	Intravenous administration	Acute: 24 h; chronic: 28 d	None	Dose- and time-dependent	21
Mice	*in vivo*	Lung	20 and 110 nm, spherical	0.1, 0.5 and 1.0 mg/kg	Inhalation	Acute: 40 h; chronic: 21 d	Pulmonary fibrosis	Size- and coating-dependent	353
Mice	*in vivo*	Kidney, liver and spleen	2.45-19.53 nm	0.37, 0.65, 13 and 21 mg/kg	Oral administration	27 d	Tissue destruction; cell necrosis and apoptosis	dose-dependent	354
Rat	*In vivo*	Brain	>100 nm	5 and 50 mg/kg	Oral administration	79 d	Cell death, disturbed neurotransmitter and cytokine production, ROS generation	--	355
Rat	*In vivo*	Sperm and testicular tissue	60-80 nm	30, 125 and 300 mg/kg	Intraperitoneal injection	28 d	Decrease normal sperm morphology, sperm vitality and sperm count	Dose-dependent	348
Rat	*In vivo*	Kidneys, liver and blood	20-65 nm	2,000 mg/kg	Intraperitoneal administration	3 d	Liver and kidney damage	Time- and dose-dependent	356
Rat	*In vivo*	Lung, spleen, liver, kidney, thymus and heart	6.3-629 nm	0.5 mg/kg	Intravenous administration	24 h	Liver and kidney damage; chromosome breakage; genotoxicity	Dose-dependent	357
Rat	*In vivo*	Epididymal sperm	20-30 nm	50, 100 and 200 mg/kg	Oral administration	90 d	Sperm anomalies; decrease sperm viability	Dose-dependent	37
Rat	*In vivo*	Brain	3-10 nm, spherical	1 and 10 mg/kg	Intragastric administration	14 d	Neuron shrinkage; cytoplasmic or foot swelling of astrocytes	Dose-dependent	38
Rats	*In vivo*	Kidney, liver and blood	20-60 nm, spherical	2,000 mg/kg bw, twice injections	Intraperitoneal injection	5 d	Liver and kidney damage; blood parameters disrupt	Dose- and time-dependent	356
Rat	*In vivo*	Spleen, liver, and lymph nodes and blood	20 nm and 100 nm	6 mg/kg	Intravenous administration	28 d	Suppression of the natural killer cell activity; stimulate LPS mitogen; increase cytokine production	Dose-dependent	358
Rat	*In vivo*	Liver and kidney	56 nm	30, 125 and 500 mg/kg	Oral administration	90 d	Liver damage; bile-duct hyperplasia	Dose- and gender-dependent	341
Rat	*In vivo*	Kidney	52.7-70.9 nm	10 ml/kg	Oral administration	90 d	Deposite in kidneys	Dose-dependent	340
Female ICR mice; male guinea pigs	*In vivo*	Oral, skin and eye	10-20 nm, spherical	5,000 mg/kg (oral); 50 and 5,000 ppm (eye); 50 and 100,000 ppm (skin)	Oral administration; eye drops; skin contact	1, 2 and 3 day	Conjunctivae irritation	—	326
Male ICR mice	*In vivo*	Blood, liver, spleen, kidney, lungs and brain	10, 40 and 100 nm, spherical	10 mg/kg	Intravenous injection	24 h	Bleeding or necrosis of multiple internal organs	Size- and tissue-dependent	331
BN and SD rats	*In vivo*	Lung	20, 110 nm, spherical	0.1 mg/kg or 90 breaths/minute	Intratracheal administration	1, 7 and 21 d	Lung eosinophilia and bronchial hyperresponsiveness; distruction of blood/alveolar epithelial permeability barrier	Dose- and size-dependent; rat strains related	46
Mice and guinea pigs	*in vivo*	Lung, lymph node, heart, liver and kidney	10-20 nm, spherical	5,000 mg/kg, 5000 ppm	Oral administration, eye and skin contact	14 d	No mortality and toxic signs		326
Freshwater fish	*In vivo*	Embryo	25.9-36.7 nm, spherical	Acute: 0.3, 0.6, 1.2, 2.4 and 4.8 mg/L; subchronic: 0.05, 0.1, 0.25 and 0.5 mg/L	Incubation	14 d	Liver damage; deplete glutathione; deactivate lactate dehydrogenase and antioxidant enzymes	Time- and dose-dependent	39
Japanese medaka	*in vivo*	Embryo	20-37 nm, spherical	0, 0.5, 1.0, 2.0, 4.0 and 8.0 mg/L	Oral administration	48 h	death	dose-dependent	327
Zebrafish	*In vivo*	Embryo	20 and 110 nm, spherical	0.08, 0.4, 2, 1,0 and 50 mg/L	Hatch	5 d	Multiple developmental abnormalities	Size- and surface coating-dependent	359
Zebrafifish	*In vitro* and* in vivo*	Brain, heart, yolk and blood of embryo	5-20 nm	5, 10, 25, 50 and 100 µg/mL	Hatch	24, 48 and 72 h	Multiple developmental abnormalities	Concentration-dependent	43
*Drosophila melanogaster*	*in vivo*	Parents, egg and offspring	2-20 nm	10, 20, 30, 40, 50 and 100 mg/L	Oral administration	5,10, 15, 30, 45, 60 and 95 min	Abdominal pigmentation	Dose-dependent	360
*Drosophila melanogaster*	*In vivo*	Germline stem cell; testis	20 nm	2, 3.5 and 5 mg/L	Oral administration	24h, 5 d	Delay the development of the F1 offsprings; ROS generation; premature GSC differentiation	Dose-dependent	42
*Caenorhabditis elegans*	*In vivo*	The worms' body	96.4±35.6 nm, spherical	0-1mg/L	Culture	6 and 24 h	DNA damage, ROS generation, inhibition of growth	Dose- and time-dependent	361
*Caenorhabditis elegans*	*In vitro*	The worms' body	< 100 nm, spherical	0.025, 0.05 and 0.075 µg/mL	Culture	24 h	ROS generation; DNA damage	Size-dependent	152
—	*In vitro*	rat brain microvascular endothelial cells, pericytes, and astrocytes	7±2 nm	1 and 10 μg/mL	Incubation	24 h	Trx system, Nr4a1 and Dusp1 regulaion , inflammation and apoptosis	Dose-dependent	338
—	*In vitro*	Mouse ESCs	20.2 ±4.1 nm, spherical	5.0 µg/ml	—	24 h	Heat shock protein and the metallothionein families regulation, induce oxidative stress and apoptosis	Dose-dependent	362
—	*In vitro*	Mouse microglia N9 cell line, N27 neuronal cells	49.7±10.5 nm, spherical	50 μg/mL	Incubation	24 h	Nitric oxide and TNFα production	Dose-dependent	363
—	*In vitro*	Mouse lymphoma cell line, human lymphoblastoid cells	20, 50 and 100 nm, spherical	0-400 μg/mL	Incubation	4, 8, and 24 h	DNA mutants	Size-, concentration- and coating-dependent	47
—	*In vitro*	Rat primary cerebral astrocytes	24.18±4.14 nm, spherical	0.01, 0.1, 1 and 10 mg/mL	Incubation	24 h	Neuroinflammation and apoptosis; increase caspase activities	Dose-dependent	337
—	*In vitro*	Primary astrocyte cell, rat glioma C6 cell line	6.9-8.7 nm, spherical	0.1, 1, 10, 50, 75 and 100 µg/mL	Incubation	24 h	Necrosis and apoptosis	Dose-dependent	364
—	*In vitro*	Murine brain ALT astrocytes, murine microglial BV-2 cells and mouse neuroblastoma Neuro-2a (N2a) cells	3-5 nm	0.5, 1, 5, 10 and 12.5µg/mL	Incubation	24 h	Cytokine secretion, Aβ amyloid deposition, inflflammatory response	Dose-dependent	339
—	*In vitro*	UMR-106	6 nm, cubic	10, 25, 50, 100, 150 and 225 μM	Incubation	24h	Decrease lysosomal and mitochondrial activity	Dose-dependent	365
—	*In vitro*	U937 cell	4,20 and 70 nm, round	1.56, 3.12, 6.25, 12.5, 25 and 50 µg/mL	Incubation	24 h	Oxidative stress; cytokines release	Size-dependent	50
—	*In vitro*	HepG2 cell line	20 nm	2.5 to 50 µg/cm^3^	Incubation	24 h	Endogenous antioxidant defence regulation	Dose-dependent	366
—	*In vitro*	Jurkat T, NCI-H460, HeLa cells, HepG2, MCF-7, Beas-2B	5-10 nm	0.2, 0.5 and 1 mg/L	Incubation	4, 12 and 24 h	DNA damage; p38 MAPK activation; cell arrest; apoptosis	Time- and concentration- dependent	27
—	*In vivo*	Human lymphocytes and sperms	8-10 nm	Density gradient: 1:9, 1:3, 1:1	—	30 and 60 mins	Cell viability decrease	Concentration- and time-dependent	367
—	*In vitro*	Pk15	61.2±33.9 nm, nonuniform	50 mg/L	Incubation	24 and 48 h	Genotoxicity in Pk15 cells	Dose-dependent	342
—	*In vitro*	Zebrafish ovarian follicle cells	30-55 nm	30 μg/mL	Incubation	2 h	Apoptosis of ovarian follicle cells; germinal vesicle breakdown	Concentration-dependent	349

*NOTE: UMR 106, rat osteosarcoma cells; MDA-MB-231, triple negative breast cancer cell line; PMBC, peripheral blood mononuclear cell; HepG2, human liver cancer cell line; Jurkat T, human T lymphocyte cell line; NCI-H460, human lung cancer cell line; MCF-7, human breast cancer cell line; Beas-2B: human bronchial epithelial cells; SPD, surfactant protein-D; U937, human histiocytic lymphoma cell line; Pk15, pig kidney cell line; BV-2, murine microglial cell line; N2a cells, mouse neuroblastoma; HEKs, human embryonic kidney cells; A549, human lung carcinoma; BxPC-3, human pancreas adenocarcinoma cells; PC3, prostate adenocarcinoma cells; HepG2, hepatocellular carcinoma cells; ESCs, embryonic stem cell; CNE, nasopharyngeal carcinoma cells; AsPC-1, pancreas adenocarcinoma cells; U-87 MG, glioblastoma cells; SW480, colorectal adenocarcinoma cells; EC109, esophageal cancer cells; VSMC, vascular smooth muscle cells; HMEC, human microvascular endothelial cells; LO2, hepatocytes; 293FT, embryonic kidney cells.
